# The impact of sex hormones and sex chromosomes on the HDL anti-inflammatory capacity in transgender individuals

**DOI:** 10.1016/j.jlr.2026.101076

**Published:** 2026-06-07

**Authors:** Ana Vankova, Veronika Tillander, Yu Lei, Margery A. Connelly, Stefan Arver, Anna Wiik, Thomas Gustafsson, Uwe J.F. Tietge

**Affiliations:** 1 Division of Clinical Chemistry, Department of Laboratory Medicine, Karolinska Institutet, Stockholm, Sweden; 2 Labcorp, Morrisville, North Carolina, USA; 3 ANOVA, Andrology, Sexual Medicine and Transgender Medicine, Karolinska University Hospital, Stockholm, Sweden; 4 Division of Clinical Physiology, Department of Laboratory Medicine, Karolinska Institutet, Stockholm, Sweden; 5 Unit of Clinical Physiology, Karolinska University Hospital, Stockholm, Sweden; 6 Clinical Chemistry, Karolinska University Laboratory, Karolinska University Hospital, Stockholm, Sweden

**Keywords:** HDL, HDL anti-inflammatory capacity, inflammation, CVD, HDL function, HDL subspecies, lipidome, transgender, sex hormones, sex chromosomes

## Abstract

Interest in the cardiovascular disease (CVD) field is shifting from high-density lipoprotein cholesterol (HDL-C) levels to exploring function of HDL particles. Among these, anti-inflammatory properties of HDL emerged as key metric associated with protection against incident CVD events, notably in a highly sexually dimorphic manner. Therefore, the present study investigated the impact of sex hormones and sex chromosomes on the HDL anti-inflammatory activity. The HDL anti-inflammatory capacity (HDL-mediated suppression of TNFα-induced *VCAM-1* mRNA expression in endothelial cells) was determined in 14 transgender men and 17 transgender women during gender-affirming hormone therapy at T0 (baseline), T1 (hormonal castration) and T12 (following 11 months of hormone substitution). HDL subspecies were characterized by nuclear magnetic resonance spectroscopy, the HDL lipidome by liquid chromatography/tandem mass spectrometry. The HDL anti-inflammatory capacity remained largely unaffected by hormonal changes. HDL-C and total HDL particle numbers did not correlate with the HDL anti-inflammatory activity but, interestingly, specific associations with distinct HDL subpopulations emerged (*P* < 0.05). Increases in HDL core lipids were related to a worse HDL anti-inflammatory function (*P* < 0.05), while increases in specific sphingomyelin, phosphatidylcholine, phosphatidylserine and phosphatidylinositol subspecies showed an opposite association (each *P* < 0.05). Combined, these data indicate that (i) neither sex chromosomes nor substantial changes in sex hormones have a fundamental impact on the HDL anti-inflammatory function and (ii) specific HDL subspecies and HDL lipids associate with the anti-inflammatory function of HDL, potentially opening an avenue to therapeutically improve HDL functionality.

Cardiovascular disease (CVD) represents the leading cause of morbidity and mortality worldwide (1). Despite considerable progress, personalized CVD risk prediction remains an unmet clinical need (2). While in large population studies circulating levels of high-density lipoprotein cholesterol (HDL-C) are inversely correlated with the risk of atherosclerotic CVD, recent pharmacological intervention trials and genetic studies failed to show a causative relationship between HDL-C and incident CVD events (3, 4). Combined, these results caused a shift of concept away from determining static HDL-C levels toward dynamic anti-atherogenic HDL function metrics (2, 5). Next to the role of HDL in mediating cholesterol efflux from macrophage foam cells and facilitating reverse cholesterol transport (6), HDL exerts prominent anti-inflammatory effects (7). These might be especially relevant given the recognition of atherosclerosis as an inflammatory disease of the vessel wall amenable to anti-inflammatory therapeutic interventions (8). We recently demonstrated that in the general population, a better HDL anti-inflammatory capacity at baseline is associated with a lower incidence of CVD events during follow-up (9). Interestingly, a significant sexually dimorphic effect was observed for this association, with women exhibiting a stronger relative protection compared to men (9). This observation ties into the broader context of certain CVD features differing substantially between the sexes (10, 11). However, the underlying basis of these effects is poorly understood, including the relative contribution of sex hormones and sex chromosomes. In addition, although initial attempts have been made to relate the HDL lipidome to functional properties of HDL particles (12), data addressing this question on a more global scale and in a sex-specific manner are still scarce. Beyond the general population, such knowledge is particularly relevant for the growing number of individuals seeking gender-affirming hormonal treatment, a group for whom CVD risk prediction remains understudied (13).

Therefore, the current work aimed to assess changes in the HDL anti-inflammatory activity, HDL subspecies distribution and the HDL lipidome in response to gender-affirming hormone therapy in a contemporary cohort of transgender individuals. Further, we addressed the potential intrinsic impact of sex chromosomes at the time point of chemical castration.

## Materials and methods

### Study population

The study was part of the single-center observational cohort study GETS (GEnder Dysphoria Treatment in Sweden, Clinical Trials identifier NCT02518009) and included a total of 31 participants (14 transgender women and 17 transgender men) undergoing gender-affirming hormone therapy. All participants were referred to Andrology, Sexual Medicine and Transgender Medicine (ANOVA), Karolinska University Hospital, Stockholm, Sweden for gender dysphoria. Detailed eligibility criteria have been published previously (14). The study protocol was approved by the Swedish Ethical Review Authority (2014/409-31/4), and all participants gave both written and oral informed consent. The research was conducted in accordance with the 1975 Declaration of Helsinki. The hormonal treatment was initiated by endogenous hormone suppression with a gonadotropin-releasing hormone (GnRH) antagonist (Degarelix 240 mg subcutaneously), bringing sex hormones to castrate levels within 24h. After 4 weeks, transgender men were treated with testosterone undecanoate intramuscularly, beginning with two 1000 mg doses six weeks apart, followed by a maintenance dose every 10th week to maintain androgen levels within the physiological adult male reference range. Every third month GnRH analogue was administered intramuscularly to maintain gonadotropin suppression. Transgender women were treated with transdermal estradiol in the form of gel (1 or 2 mg daily) or patches (100–200 μg every 24h). A few cases received intramuscular estrogen polyphosphate (80 mg IM every 2–4 weeks).

### Biochemical blood analysis

Following a 5-min rest, blood was drawn via antecubital venipuncture. Blood samples were available at baseline (T0), 4 weeks into the study, at the time point of chemical castration (T1) and at 12 months, following 11 months of gender-affirming hormone treatment (T12). Total cholesterol, HDL-C, triglycerides, fasting insulin, fasting glucose and high-sensitivity C-reactive protein (hsCRP) were analyzed at the Karolinska University Hospital Laboratory using standard clinical chemistry methods and reagents (Roche). Low-density lipoprotein cholesterol (LDL-C) was calculated using the Friedewald equation. Sex hormone quantification was performed using liquid chromatography coupled with mass spectrometry to determine estradiol and testosterone concentrations. Homeostatic Model Assessment for Insulin Resistance (HOMA-IR) was calculated as HOMA-IR = (fasting insulin ∗ fasting glucose)/22.5.

### HDL anti-inflammatory activity

The HDL anti-inflammatory activity was assessed in vitro as previously described (9, 15). Plasma samples were kept at −80°C until analysis. HDL was isolated from plasma by the precipitation of apoB-containing lipoproteins using 36% polyethylene glycol (PEG 6000; Sigma) (9, 15, 16). Human umbilical vein endothelial cells (HUVECs, provided by the Endothelial Cell Core Facility of the University Medical Center Groningen) were grown on collagen pre-coated T75 cell culture flasks. The cells were then seeded in 96 well plates (10,000 cells/well) and pre-incubated for 30 min with either 2% of the ApoB-depleted plasma or an equal volume of PEG in phosphate buffered saline (PBS) as a control. PEG-containing controls were treated identical to the apoB-depleted plasma samples including centrifugation. Subsequently, the cells were stimulated with 5 ng/ml recombinant human tumor necrosis factor α (TNFα, PHC3011, Thermo Fisher) for 5 h. The cells were lysed and the mRNA was reverse transcribed to cDNA. *VCAM-1* (vascular cell adhesion molecule-1) expression levels were determined by quantitative real-time polymerase chain reaction (Bio-Rad CFX96) as described (6, 14). The data are presented as a fold change of *VCAM-1* expression relative to the TNF-α only treated control samples. Thus, lower *VCAM-1* expression values given as fold change indicate a higher anti-inflammatory activity of HDL. In validation experiments, we first assessed in n = 8 healthy controls the contribution of HDL within apoB-depleted plasma to the overall anti-inflammatory activity (Supplementary Fig. S1A). When HDL was isolated out of apoB-depleted plasma using ultracentrifugation the volume-adjusted infranatant retained anti-inflammatory activity, however, substantially less than in apoB-depleted plasma containing HDL. Further, we compared apoB-depleted plasma with HDL isolated by ultracentrifugation, adjusting for cholesterol concentrations (Supplementary Fig. S1B). HDL obtained by ultracentrifugation exhibited overall slightly lower anti-inflammatory activity in the side-by-side comparison with apoB-depleted plasma. Given that all the samples used in the present work were from young, healthy adults without significant systemic inflammation, these results would be consistent with a concept that small pre-beta HDL that are contained within apoB-depleted plasma but are, at least partially, lost in HDL isolation by ultracentrifugation contribute to the overall anti-inflammatory activity of apoB-depleted plasma.

### HDL subclass analysis

HDL subclass analysis was performed using nuclear magnetic resonance (NMR) spectroscopy at Labcorp (Morrisville, NC) using EDTA plasma samples (17). HDL particle numbers of the different subclasses were characterized as H1P through H7P, with specific group-defining diameter measurements of 7.4 nm (H1P), 7.8 nm (H2P), 8.7 nm (H3P), 9.5 nm (H4P), 10.3 nm (H5P), 10.8 nm (H6P), and 12.0 nm (H7P). Hereby small HDL corresponds to H1 and H2 particles, medium HDL to H3 and H4 particles, and large HDL encompass H5 through H7 particles. The total HDL particle concentration (HDL-P) was calculated as the sum of H1P to H7P.

### Targeted HDL lipidomics

The samples were prepared by a modified Folch extraction (18). Twenty μL of ApoB-depleted plasma was diluted with 0.9% saline up to a final volume of 200 μl. One mL of methanol containing 0.5 μg/ml of each internal standard (Supplementary Table S1) was added to individual samples, followed by 2 ml of chloroform. The samples were vortexed and incubated at room temperature for 10 min prior to adding 400 μl of 0.9% saline and mixing the samples again. To yield a good phase separation, the samples were centrifuged at 2000 x g at 4°C for 6 min. The organic phase was carefully transferred to new glass tubes and was evaporated to dryness under a stream of nitrogen. Lipids were then dissolved in 1 part hexane and 2 parts isopropanol to a final volume of 100 μl and transferred to vials for LC-MS/MS analysis.

Lipidomic analysis was performed on a Waters Acquity ® UHPLC system coupled to a Xevo® TQ-X quadropole (Waters). The samples were separated on an ACQUITY UHPLC C18 column (150 mm, 2.1 mm 1.7 μm) set to a temperature of 60°C. The mobile phase consisted of 10 mM ammonium formate and 0.1% (v/v) formic acid in 60% acetonitrile (mobile phase A) and 10 mM ammonium formate and 0.1% (v/v) formic acid in 10% (v/v) acetonitrile and 90% (v/v) isopropanol (mobile phase B).

A constant flow of 0.200 ml/min mobile phase was applied over the column. A gradient program was set as follows; 0–2.25 min a 99% mobile phase A and 1% B was gradually changed to 30% B, further changed from 30% to 51% B up to 3.75 min, 51% - 99% B up to 14.06 min, following a constant flow of 99% B up to 14.25 min, and then a decrease of B to starting levels of 1% until 16.88 min.

The LC-separated molecules were ionized by a UniSpray (Impactor voltage 2.0 kV) in either positive or negative ion mode depending on the lipid species (Supplementary Table S1). Nitrogen gas was used as desolvation gas at 1000 L/h and cone gas at a flow rate of 150 ml/min. Argon was used as collision gas at 0.15 ml/min.

Sample cone voltage was set to either 30 V for all phospholipids and triacylglycerols, or 50 V for cholesteryl esters. The collision energy voltage was adjusted depending on the lipid group and expected fragmentation pattern of the lipids, determined by fragmentation patterns of lipid standards, ranging between 16 to 24 eV (Supplementary Table S1).

Semi-quantitative analyses were based on calibration curves in six different concentrations, in which each lipid standard response (peak area of the lipid standard to the area of each appropriated internal standard, 0.5 μg per sample) was plotted against the standard lipid concentration in w/v. The Waters Acquity ® UHPLC - Xevo® TQ-X was controlled, data acquired and further quantified using MassLynx™ software.

To assure that using this method on apoB-depleted plasma results in a representative determination of HDL-associated lipids, we isolated the HDL fraction (d < 1.21) from apoB-depleted plasma of 6 healthy controls (3 males and 3 females) by KBr-based ultracentrifugation. This way we confirmed that 93.4% of phosphatidylcholines, 92.5% of sphingomyelins, 97.7% of cholesteryl esters, 78.0% of triacylglycerols, 97.1% of alkenylphosphatidylcholines, 78.0% of phosphatidylserines and 97.6% of phosphatidylethanolamines were associated with the HDL fraction. A detailed overview of all specific lipid classes and species is provided as Supplementary Fig. S2 in the online supplement.

### Statistical analysis

To explore any changes among time points (T0, T1 and T12), Wilcoxon matched-pairs signed rank test was used, while for differences between groups (transgender women and transgender men) the Mann-Whitney U-test was used. Kruskal-Wallis test was performed to test changes within groups for baseline clinical parameters and individual lipid species in lipidomics analyses followed by post-hoc Dunn's test. To investigate associations between clinical parameters, total lipid groups and specific lipid species with *VCAM-1* expression, Spearman correlation analysis was performed. Although the number of tested features was relatively limited and the nature of this analysis was exploratory with the aim of avoiding an overly conservative approach that could obscure potentially relevant associations, we also provide false discovery rate (FDR) correction for multiple hypothesis testing using the Benjamini-Hochberg method. A *P*-value lower than 0.05 was considered statistically significant. All tests and figures were done in GraphPad Prism (version 10.5.0, Dotmatics, Boston, MA), apart from the lipidomics Kruskal-Wallis analysis where Python was used.

The present study was primarily exploratory in nature, given the inherent practical challenges associated with recruitment and longitudinal follow-up of a well-defined transgender cohort. Based on an intra-assay coefficient of variation (CV) of 7.6% and an inter-assay CV of 8.8% (9), the combined analytical imprecision of the HDL anti-inflammatory assay, the main outcome variable of the study, is approximately 11.6%. Under a simplified assay-limited model, this corresponds to a minimum detectable paired mean change of about 11.2% in the group of transgender women and 12.3% in transgender men (two-sided alpha 0.05, 80% power).

## Results

### Baseline characteristics of the participants

The clinical characteristics of the cohort are shown in Table 1. At baseline (T0), before hormone treatment initiation, the average age of transgender men was 25.3 years, while transgender women were on average 26.5 years old. The body mass index (BMI) of both transgender men and transgender women was within a healthy range at baseline (transgender men 24.4 kg/m^2^ and transgender women 22.1 kg/m^2^) as well as at the end of the study period (transgender men 22.9 kg/m^2^ and transgender women 22.7 kg/m^2^). Most of the clinical parameters remained stable except for HDL cholesterol which decreased in transgender men from T1 to T12 (*P* = 0.03) as shown in Table 1. Interestingly, systemic inflammation as reflected by hsCRP levels, increased in transgender men at T12 compared to the T0 time point (*P* = 0.027). As expected, circulating estrogen and testosterone levels showed substantially higher estrogen in cis- and transwomen and significantly higher plasma testosterone in cis- and transmen (Supplementary Fig. S3). Systolic and diastolic blood pressure were within the normal range for both groups.

**Table 1:** Clinical characteristics of study participants.

	Transgender men (n = 14)				Transgender Women (n = 17)			
	T0	T1	T12	*P*-value	T0	T1	T12	*P*-value
Age (years)	25.3 ± 4.9				26.5 ± 3.8			
Body weight (kg)	67.6 ± 20.4	66.8 ± 19.0	69.4 ± 13.2	0.65	70.7 ± 9.9	70.5 ± 11.6	73.0 ± 9.2	0.51
BMI (kg/m^2^)	24.4 ± 7.2	23.8 ± 6.7	24.7 ± 7.7	0.44	22.0 ± 3.1	21.8 ± 3.6	22.7 ± 2.7	0.56
SBP (mm Hg)	114 ± 11	120 ± 13	123 ± 12	0.07	116 ± 10	119 ± 12	117 ± 12	0.91
DBP (mm Hg)	68 ± 9	71 ± 12	71 ± 8	0.47	70 ± 6	72 ± 9	70 ± 8	0.92
Fasting insulin (mIE/ml)	10.2 ± 5.9	10.5 ± 7.1	9.7 ± 6.1	0.96	8.2 ± 3.9	7.4 ± 2.8	10.3 ± 6.7	0.35
Fasting glucose (mmol/L)	5.1 ± 0.3	5.1 ± 0.4	5.3 ± 0.3	0.32	5.2 ± 0.3	5.2 ± 0.3	5.1 ± 0.2	0.34
HOMA-IR	4.0 ± 0.6	4.3 ± 0.6	3.9 ± 0.7	0.94	2.4 ± 1.0	2.5 ± 0.8	1.9 ± 1.1	0.50
LDL-C (mmol/L)	2.1 ± 0.8	3.7 ± 6.0	2.2 ± 0.7	0.75	2.1 ± 0.6	2.2 ± 0.8	2.0 ± 0.5	0.76
HDL-C (mmol/L)	1.6 ± 0.5	1.7 ± 0.5	1.2 ± 0.3	**0.03**	1.4 ± 0.3	1.4 ± 0.3	1.4 ± 0.3	0.88
Total cholesterol (mmol/L)	4.0 ± 0.6	4.3 ± 0.6	3.9 ± 0.7	0.23	3.8 ± 0.7	4.1 ± 0.8	3.7 ± 0.6	0.47
HDL-P (μmol/L)	16.1 ± 2.1	17.1 ± 2.2	16.3 ± 2.4	0.63	16.5 ± 2.2	16.6 ± 2.5	16.3 ± 1.5	0.94
Triglycerides (mmol/L)	0.8 ± 0.4	0.8 ± 0.5	1.0 ± 0.5	0.25	0.8 ± 0.3	0.9 ± 0.8	0.8 ± 0.4	0.92
hsCRP (mg/L)	0.8 ± 0.7	n.d.	2.6 ± 3.1	**0.03**	0.5 ± 0.4	n.d.	0.3 ± 0.2	0.23

Data are presented as means ± SD. Differences were assessed using the Kruskal-Wallis test followed by post-hoc Dunn's test or in the case of hsCRP the Wilcoxon paired test to compare the two time points. T0, baseline; T1: following 1 month of gonadal suppression treatment, when sex hormones were at castration level; T12: after 11 months of gender-affirming treatment. BMI, body mass index; SBP, systolic blood pressure; DBP, diastolic blood pressure, HOMA-IR, homeostatic model assessment of insulin resistance; LDL-C, low density lipoprotein cholesterol; HDL-C, high density lipoprotein cholesterol; hsCRP, high sensitivity C – reactive protein; n.d., not determined. Statistically significant differences (*P* < 0.05)- are indicated in bold font.

### Sex hormones leave the HDL anti-inflammatory activity largely unaffected

**Fig. 1:**
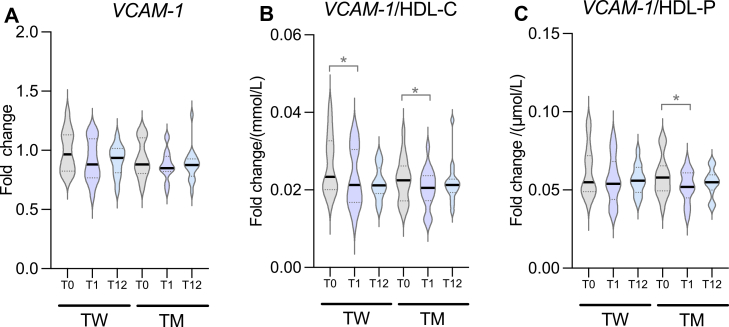
The HDL anti-inflammatory activity in response to gender-affirming hormone treatment. T0, baseline; T1, following 1 month of gonadal suppression treatment, when sex hormones were at castration level; T12, after 11 months of gender-affirming treatment. A: Fold change of *VCAM-1* mRNA expression induced by HDL relative to maximum induction by TNF-α as detailed in methods. B: Fold change of *VCAM-1* mRNA expressed relative to high density lipoprotein cholesterol (HDL-C) levels. C: Fold change of *VCAM-1* mRNA expressed per particle of HDL (HDL-P). TW, transgender women; TM, transgender men. ∗*P* < 0.05.

A potential change in the HDL anti-inflammatory function during gender-affirming hormone therapy was evaluated using TNFα-induced *VCAM-1* expression in HUVECs as read-out. Hereby, lower *VCAM-1* expression values reflect a better HDL anti-inflammatory activity. Interestingly, despite drastic changes in hormone levels, the HDL anti-inflammatory activity did not show significant changes in either transgender men or women (Fig. 1A). However, there was a small but significant increase in the HDL anti-inflammatory activity in both, transgender women and transgender men, at castration level (T1) compared with baseline (T0) when the HDL anti-inflammatory activity was adjusted for HDL cholesterol (Fig. 1B, *P* = 0.013 (transgender women), *P* = 0.030 (transgender men)), while when expressed per HDL particle this was only seen in transgender men (Fig. 1C, *P* = 0.04). These results indicate that the HDL anti-inflammatory function is generally stable and not to a major extent determined by within person changes in androgen and estrogen levels.

### Sex hormones influence the HDL subspecies distribution

**Fig. 2:**
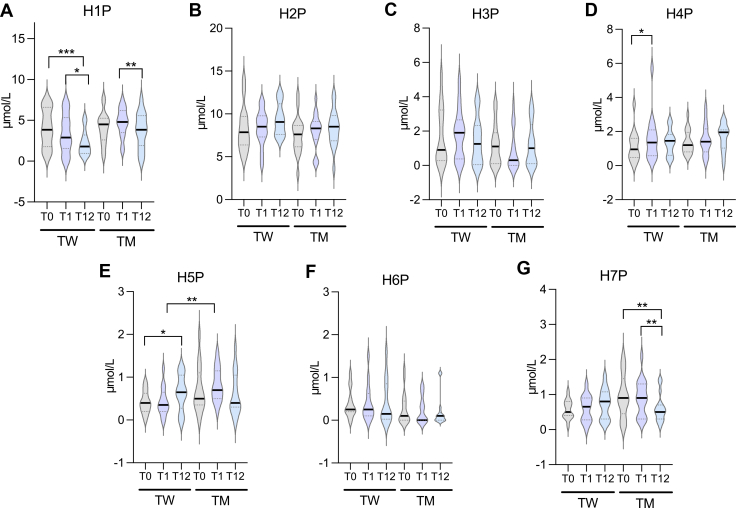
Changes in HDL subspecies in transgender women (TW) and transgender men (TM) during the course of gender-affirming hormone therapy. Plasma levels of HDL subspecies sorted from smallest (H1P) to largest (H7P) were determined by nuclear magnetic resonance spectroscopy as detailed in methods. A: H1P, B: H2P, C: H3P, D: H4P, E: H5P, F: H6P, G: H7P. T0, baseline; T1,: following 1 month of gonadal suppression treatment, when sex hormones were at castration level; T12,: after 11 months of gender-affirming treatment. ∗*P* < 0.05, ∗∗*P* < 0.01 and ∗∗∗*P* < 0.001.

Further, the impact of sex hormones and sex chromosomes on the HDL subspecies distribution was investigated (Fig. 2). The smallest H1P particles (Fig. 2A) decreased in transgender women from baseline to T12 (*P* < 0.001) and from T1 to T12 (*P* = 0.013). They also decreased significantly from T1 to T12 in transgender men (*P* = 0.001). H4P particles increased from baseline to T1 (*P* = 0.046, Fig. 2D) in transgender men, while H5P particles (Fig. 2E) increased from baseline to T12 (*P* = 0.035) in transgender women and were also higher in transgender men compared to transgender women at T1 (*P* = 0.005). H7P were decreased at T12 in transgender men, both compared to T0 and T1 (*P* = 0.008, and *P* = 0.004 respectively, Fig. 2G), while the reciprocal increase in transgender women was not significant.

**Fig. 3:**
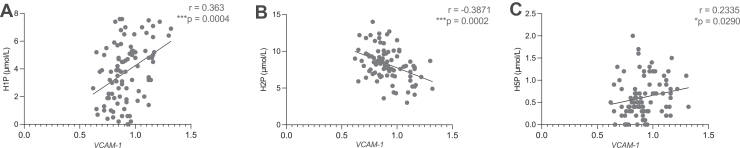
Correlations of distinct HDL subspecies with relative *VCAM-1* expression in all study participants combined. Spearman correlation analysis of *VCAM-1* mRNA expression and the respective plasma concentrations of HDL subspecies determined by nuclear magnetic resonance spectroscopy as detailed in methods; lower numbers indicate smaller particle sizes. (A) H1P, (B) H2P, (C) H5P. Correlation coefficients and statistical significances are given.

To further investigate a potential association between different HDL-subclasses with the HDL anti-inflammatory capacity, *VCAM-1* expression was correlated with the concentrations of the different HDL-subclasses across different time points and participant groups. Higher plasma concentrations of the smallest HDL particles (H1P) were significantly associated with a worse HDL anti-inflammatory activity (*P* = 0.0004, Fig. 3A). Interestingly, the opposite was found for the slightly larger H2P subclass, where increased concentrations of H2P were related to a better anti-inflammatory activity (*P* = 0.0002, Fig. 3B). In addition, H5P HDL particles showed a somewhat weaker but significant correlation with worse anti-inflammatory activity (*P* = 0.0290, Fig. 3C). No such correlations were observed for the other HDL subclasses (H3P, r = −0.121, *P* = 0.258, Fig. 3C; H4P, r = 0.033, *P* = 0.758, Fig. 3D; H6P, r = −0.029, *P* = 0.7872, Fig. 3F; H7P, r = 0.137, *P* = 0.198, Fig. 3G). These data suggest that different HDL subspecies differ in their contribution to the overall anti-inflammatory activity of HDL.

### Sex hormones do not cause major shifts in the HDL lipidome

**Fig. 4:**
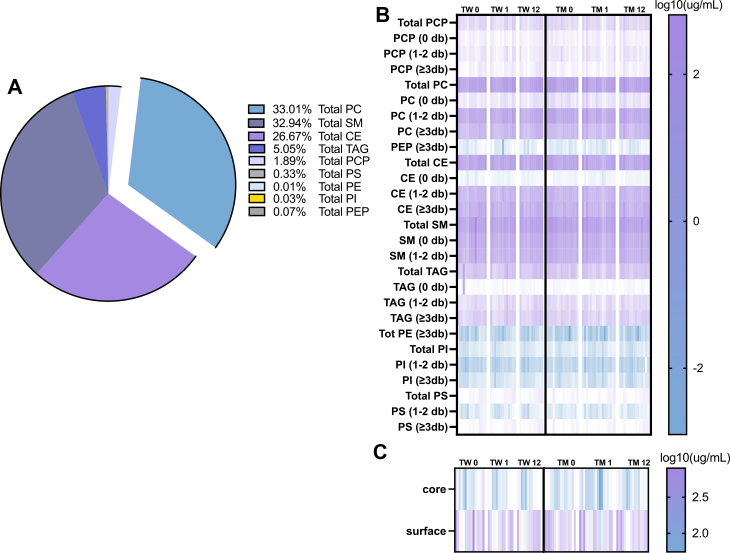
The HDL lipidome remains largely unaffected by sex hormones and sex chromosomes. Lipidomics analyses were performed using liquid chromatography-tandem mass spectrometry as detailed in methods. A: Overall distribution of the main lipid groups within HDL based on the average total concentrations of all participants combined. B: Levels of distinct lipid species grouped by saturation level (data are shown on a log10 scale). C: Distribution of HDL lipid levels separated into HDL core and surface lipids (data are shown on a log10 scale). T0, baseline; T1, following 1 month of gonadal suppression treatment, when sex hormones were at castration level; T12, after 11 months of gender-affirming treatment; TW, transgender women; TM, transgender men; PC, phosphatidylcholines; SM, sphingomyelins; CE, cholesteryl esters; TAG, triacylglycerols; PCP, alkenylphosphatidylcholines; PS, phosphatidylserines; PE, phosphatidylethanolamines; PI, phosphatidylinositols; PEP, alkenylphosphatidylethanolamines; db, double bond.

The HDL particle carries a diverse lipid cargo comprising different lipid groups and species (Fig. 4A), both apolar (core) and polar (surface) which have been related to its function (12, 19). Using targeted lipidomics of over 300 lipid species we first aimed to explore the impact of sex hormones and sex chromosomes on the HDL lipidome. Overall, we observed no significant changes among the participants (Fig. 4B). A more detailed representation of the distribution by lipid group is shown in Supplementary Fig. S4, while individual lipid species are given in Supplementary Fig. S5. No changes were detected when the lipids were classified based on their location on the HDL particle (core vs. surface) (Fig. 4C).

**Fig. 5:**
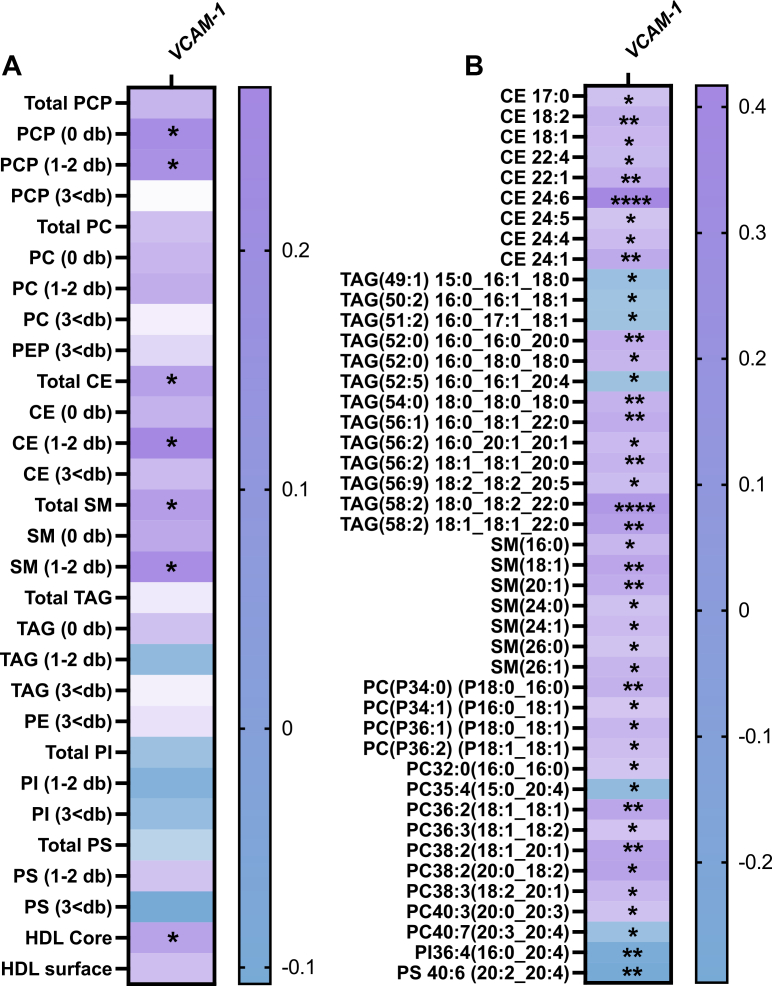
Correlations of specific HDL lipid groups and lipid species with relative *VCAM-1* expression in all study participants combined. Correlation of relative *VCAM-1* mRNA expression with (A) lipid groups classified based on saturation level and (B) individual lipid species. PC, phosphatidylcholines; SM, sphingomyelins; CE, cholesteryl esters; TAG, triacylglycerols; PCP, alkenylphosphatidylcholines; PS, phosphatidylserines; PE, phosphatidylethanolamines; PI, phosphatidylinositols; PEP, alkenylphosphatidylethanolamines; db, double bond. Heatmap colors represent correlation coefficient (r) values from Spearman analysis, significance is indicated as ∗*P* < 0.05, ∗∗*P* < 0.01, ∗∗∗∗*P* < 0.0001. Following FDR correction, significance was retained for CE 24:6 (q = 0.01) and TAG(58:2) 18:0_18:2_22:0 (q = 0.05).

To further investigate which HDL lipids might be relevant as potential mediators of the HDL anti-inflammatory activity, lipids were first summarized as general lipid categories as well as grouped into HDL-core lipids, composed of the neutral cholesteryl esters (CE) and triacylglycerols (TAG) and HDL-surface lipids including the remaining polar lipid species. Correlation analysis of the lipid species on HDL and the *VCAM-1* mRNA expression revealed that the sum of HDL-core lipid components was positively correlated to *VCAM-1* expression indicating a lower HDL anti-inflammatory activity with an enrichment of HDL with these hydrophobic lipids (*P* = 0.045) (Fig. 5A). The major driver of this association seemed to be the content of cholesteryl esters with C18:1 or C18:2 acyl-chains, next to other less abundant CEs. While the total amount of TAGs was not correlated with *VCAM-1* expression (Fig. 5A), a few low abundant TAG species associated positively with *VCAM-1* (Fig. 5B). The sum of the HDL-surface lipids did not significantly associate with *VCAM-1* expression (Fig. 5A). On the other hand, enrichments of phosphatidylcholines (both ester lipids, PC, and their corresponding ether lipids, PCP) with monounsaturated acyl-chains, and sphingomyelins with saturated or monounsaturated acyl-chains, correlated positively with *VCAM-1* expression (Fig. 5B). Interestingly, the only lipid species that showed a significant negative association with *VCAM-1* expression and thus with improved HDL anti-inflammatory function were phospholipids (and one TAG species) containing arachidonic acid (C20:4) in their structure. Following FDR correction, significance was retained for CE 24:6 (q = 0.01) and TAG(58:2) 18:0_18:2_22:0 (q = 0.05).

## Discussion

The present work demonstrates that, under the applied experimental conditions, several critical aspects of HDL are remarkably resilient to rather substantial changes in the individual hormonal environment. The HDL anti-inflammatory function, a key anti-atherosclerotic metric of HDL particles (9), together with the HDL subparticle distribution and the HDL lipidome did not display major shifts during gender-affirming hormone treatment of transgender men or women including the period of chemical castration. These results hold significant positive implications for the transgender population and provide broader novel insights into the (patho)physiology of HDL function and HDL particle remodeling.

Sex differences in incidence, pathophysiology and presentation of atherosclerotic CVD have long been recognized but remain as of yet still incompletely understood (20). A similar reasoning applies to the CVD risk associated with gender-affirming hormone treatment. Summarizing current knowledge, transgender women appear to have an increased myocardial infarction risk when compared with cisgender women but not cisgender men (21, 22, 23). Of note, particularly older studies on this topic are confounded by the use of ethinyl estradiol in transgender women which has strong intrinsic adverse cardiometabolic effects (22, 23), and has not been used in the individuals included in the present study. On the other hand, transgender men are also ascribed an increased CVD risk, at least in comparison with cisgender women (21), but possibly also with cisgender men (23). Our results show that an impaired HDL anti-inflammatory activity is conceivably not a contributing factor to the apparent increased CVD risk in the transgender population, at least early in life in the young healthy participants included in our work. Further studies would be required to determine if this extends into a disease context with increasing age when more CVD risk factors that could impact HDL function weigh in.

HDL particles carry, next to cholesterol, a wide variety of potentially bioactive lipid cargo (24). Especially the group of phospholipids has been highlighted in this respect and here the particular focus has been on the anti-inflammatory biological activities of phosphatidylserines (25). We could not confirm an association between the overall phosphatidylserine content of HDL and its anti-inflammatory function. However, increased HDL levels of a particular phosphatidylserine species, PS 40:6 (20:2_20:4) that carries arachidonic acid, were correlated with a better anti-inflammatory activity, a finding that might be worth to investigate in follow-up studies, also including recombinant HDL particles. Broadening this observation, a particularly interesting result from our HDL lipidomic analysis was that altogether arachidonic acid in phospholipids (apart from one TAG species) showed a significant correlation with an improved HDL anti-inflammatory function. In general, arachidonic acid is associated with the production of pro-inflammatory mediators such as prostaglandins (PGE2), thromboxanes (TXA2) and leukotrienes (LTB4) (26). However, the production of arachidonic acid-derived signaling molecules is highly context dependent and also anti-inflammatory lipoxins (LXA4/B4) can be generated via 5-lipoxygenase and 15-lipoxygenase pathways (26); alternatively, cyclopentenone prostaglandins can be formed that activate PPAR-ɤ and thereby inhibit the NFKB pathway (27). Upstream of mediator synthesis, however, is the liberation of the fatty acid precursors that are esterified within phospholipids, mainly via the action of specialized site-specific phospholipases. Endothelial lipase, an sn-1 specific phospholipase from the lipoprotein lipase family, is for example expressed in the vessel wall and has been demonstrated to generate PPARα ligands from HDL phospholipids (28). Consequently, VCAM-1 expression is significantly downregulated resulting in the decreased adhesion of monocytes (28). Here also other PUFAs could come into play as some of these are particularly strong ligands for PPARα (29). On the other hand, secretory phospholipases with sn-2 specificity (sPLA_2_), such as group IIA or group V sPLA_2_, are also active in the vessel wall, are themselves subject to regulation by inflammatory stimuli and use HDL particles as substrate (30). sPLA_2_-generated lipid mediators could thus provide additional regulatory cues. Interestingly, both endothelial lipase and the sPLA_2_ enzymes were indicated to be regulated in a sexually dimorphic manner (31, 32, 33, 34). Beyond the current work, more mechanistic studies will be required to further delineate the complex, highly context-dependent signaling networks within the vessel wall that are suggested by such a concept.

Another relevant aspect, particularly regarding a potential therapeutic exploitation, is the question how arachidonic acid-containing phospholipids could be incorporated into HDL. A possible route would be to generate recombinant HDL particles and use specific phospholipids in the process; it remains to be seen if such an approach bears therapeutic efficacy. Since in ABCA1-mediated HDL formation cell membrane phospholipids from microdomains are used (35), enriching membrane phospholipids with arachidonic acid-containing species e.g. via dietary manipulations could represent a feasible approach. This appears particularly attractive, since the majority of HDL originates from liver (an estimate by mouse studies is 70% (36)), with the remainder being generated by the small intestine and by apoA-I complexed with phospholipids that derive from the hydrolysis of large triglyceride-containing lipoproteins (5). However, current concepts assume that mainly phospholipids from the outer leaflet are used for ABCA1-mediated HDL formation, while arachidonic acid is more enriched in phospholipid species contained within the inner leaflet (phosphatidylethanolamine, phosphatidylinositol, and phosphatidylserine) (35). Another way phospholipids are incorporated into HDL particles is via the phospholipid transfer protein (PLTP) but PLTP is rather unselective in the choice of phospholipids (37), which makes PLTP-based manipulations an approach less likely to be successful.

In addition to lipids, HDL carry a large number of non-lipid components; proteins and miRNAs are important to consider in this context (38, 39). These were not determined in the present work but might have nevertheless contributed to the presented findings. However, to date surprisingly little is known on the impact of sex on the HDL proteome. We could not identify a study that was designed to address this particular question. Part of an explanation might be offered by the high intraindividual variation of the HDL proteome, although the within-person variation appears to be limited even in disease contexts (40, 41). This point warrants future research.

Part of the motivation to conduct the current study came from our previous work demonstrating a strong sexual dimorphism with respect to the disparate meaning of HDL anti-inflammatory function results in men and women in the general population; women appeared to display a stronger protective effect (9). Our results now strongly suggest that this association is not based per se in alterations of HDL function or lipid composition due to hormonal differences. Rather, more complex sex-specific interactions between HDL and the vessel wall or the plaque microenvironment seem to play a role such as the above hypothesized regulation of the temporo-spatial balance between endothelial lipase and group IIA and group V sPLA_2_ expression. Both phospholipases and the release of site-specific lipids in the vessel wall could determine if pro- or anti-inflammatory metabolites are produced with HDL-derived fatty acids as substrate. These and other sex-specific alterations in the vessel wall that become more prominent with ageing and change with exposure to risk factors such as shear stress, modified LDL accumulation et cetera could be very important (2). Therefore, our current results support the importance of the vessel wall environment that circulating HDL particles encounter and interact with, additive to and beyond the mere composition of HDL particles.

Regarding HDL subspecies, higher levels of H1P and H5P were correlated with a worse anti-inflammatory function of HDL, while specifically higher H2P concentrations associated with a better anti-inflammatory function. Given the close size proximity of H1P and H2P particles, this result is remarkable and indicates that distinct HDL particle subspecies differ in their impact on the total anti-inflammatory activity of HDL. Comparable data in literature are scarce, however, some previous work indicated that the H2P subgroup is the most abundant one in plasma and that higher levels of H2P were associated with an increase in incident diabetes in the general population (17) as well as with decreased incident myocardial infarction and ischemic stroke in a multiethnic pooled cohort study (42). Other work directly testing the impact of HDL of different sizes on inflammation-induced adhesion molecule expression in endothelial cells concluded that smaller HDL particles have a better anti-inflammatory function (43, 44), however, it is difficult to directly compare these studies to the NMR-based sizing of HDL subspecies.

Several methodological considerations are also important for contextualizing the presented findings. Currently, there is no generally accepted standard for the isolation of HDL particles for HDL function assays; every method has certain advantages and disadvantages (5). Classical ultracentrifugation-based methods are time consuming, usually do not capture small pre-beta HDL and can affect HDL composition by the application of centrifugal forces and the ionic strengths of the solutions used to adjust densities such as KBr (5). FPLC-based methods on the other hand can capture even small HDL particles and preserve protein composition but are also laborious, can suffer from overlap with small LDL and require concentration of the very dilute HDL recovered from the columns. Taken together, both of these methods are practically not very suitable for use in larger cohorts of individuals, mainly because of the required isolation times that, at best, result in very uneven cold storage times of the isolated HDL. Therefore, the use of apoB-depleted plasma has been adopted as a faster isolation alternative (9, 16, 45, 46) that captures also small HDL species but has the disadvantages of retaining PEG, which could potentially affect conformation, solubility and function of proteins, as well as many non-HDL plasma components. However, regarding the latter point one could argue that plasma is the matrix in which the vessel wall, i.e. endothelial cells in the first line, will encounter HDL particles. Subsequently, apoB-depleted plasma was used in all large prospective HDL function studies published to date (9, 16, 45, 46). Therefore, we also continued to use this method in the current work. To assure that the results give at least a good approximation of HDL effects at large we conducted a number of control experiments demonstrating that (i) the anti-inflammatory activity of apoB-depleted plasma closely reflects the values obtained with HDL isolated by ultracentrifugation; the observed differences likely reflect the loss of small pre-beta HDL during ultracentrifugation, (ii) apoB-depleted material captures the HDL lipidome well, although lipids can distribute to a variable extent among plasma proteins. Importantly though, we want to stress that our results were obtained in young healthy individuals and that this reasoning does conceivably not apply to clinical conditions such as sepsis, in which substantial remodeling of the plasma protein composition due to pro-inflammatory cytokines and positive as well as negative acute phase proteins occurs.

Strengths of the current study are the thorough follow-up, the deep phenotyping of the participating individuals and the inclusion of a castration time point, at which the impact of sex chromosomes without interference by sex hormones can be assessed. Potential limitations are the relatively small number of included individuals that were all White and from a resource-high health care setting. However, individual socioeconomic status information was not available. Performing targeted lipidomics is on the one hand a strength, since it provides thorough quantification, but on the other hand also a limitation since global coverage, such as it is possible with untargeted methods, is not provided.

In summary, the present study demonstrates that even substantial changes in the sex hormonal environment do not translate into major effects on the HDL anti-inflammatory function, the HDL subparticle distribution and the HDL lipidome. Thereby our results indicate that these parameters are considerably stable in young, healthy adults. Conceivably, a disease context, either systemically or locally via interaction with an atherosclerosis-prone vessel wall environment, is required to remodel HDL particles and change the HDL anti-inflammatory function or its clinical meaning in a sex-specific fashion.

## Data availability

The dataset analysed in the current study is available from the corresponding author upon reasonable request.

## Supplemental data

This article contains supplemental data.

## Conflict of interest

The authors declare the following financial interests/personal relationships which may be considered as potential competing interests:

M. A. C. is an employee of and holds stock in Labcorp.
