# Supplementary material for: The impact of sex hormones and sex chromosomes on the HDL anti-inflammatory capacity in transgender individuals
Source: J Lipid Res. 2026 Jun 7;67(7):101076. doi: 10.1016/j.jlr.2026.101076 (PMC13377147; doi:10.1016/j.jlr.2026.101076)
Supplement: Supplemental Tables and Figures [file mmc1.docx]

**Supplementary material to:**

**The impact of sex hormones and sex chromosomes on the HDL anti-inflammatory capacity in transgender individuals**

Ana Vankova^1^, Veronika Tillander^1^, Yu Lei^1^, Margery A. Connelly^2^, Stefan Arver^3^, Anna Wiik^4^, Thomas Gustafsson^4,5^, Uwe J.F. Tietge^1,6^

^1^ Division of Clinical Chemistry, Department of Laboratory Medicine, Karolinska Institutet, Stockholm, Sweden

^2^ Labcorp, Morrisville, NC 27560, USA

^3^ ANOVA, Andrology, Sexual Medicine and Transgender Medicine, Karolinska University Hospital, Stockholm, Sweden

^4^ Division of Clinical Physiology, Department of Laboratory Medicine, Karolinska Institutet, Stockholm, Sweden

^5^ Unit of Clinical Physiology, Karolinska University Hospital, S-141 83 Stockholm, Sweden.

^6^ Clinical Chemistry, Karolinska University Laboratory, Karolinska University Hospital, Stockholm, Sweden


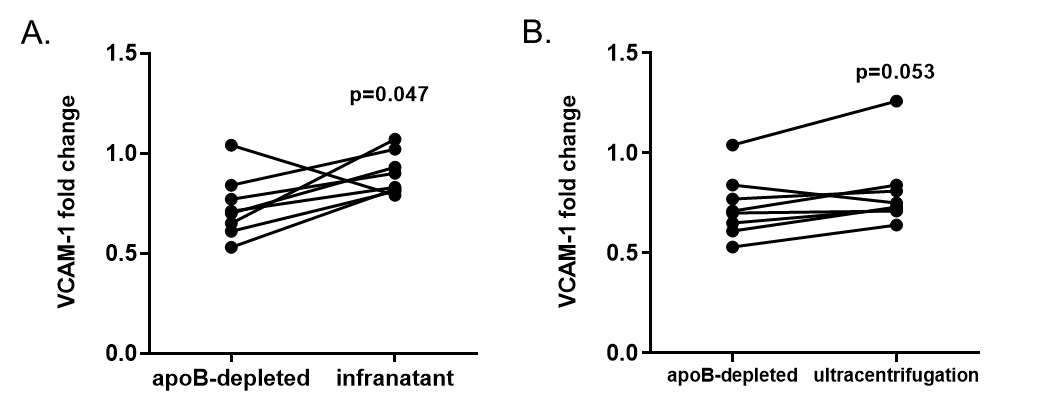


**Figure S1. Comparison of different sample inputs for the HDL anti-inflammatory assay.** (A) Comparison between apoB-depleted plasma before and after HDL particles were isolated out using KBr-based ultracentrifugation (d=1.21 g/mL). (B) Comparison between apoB-depleted plasma and HDL isolated from plasma samples by KBr-based ultracentrifugation (d=1.21 g/mL). The anti-inflammatory assay was carried out as detailed in methods using plasma samples from n=8 healthy controls. Assay input was adjusted to volume in (A) and to HDL cholesterol concentration in (B).


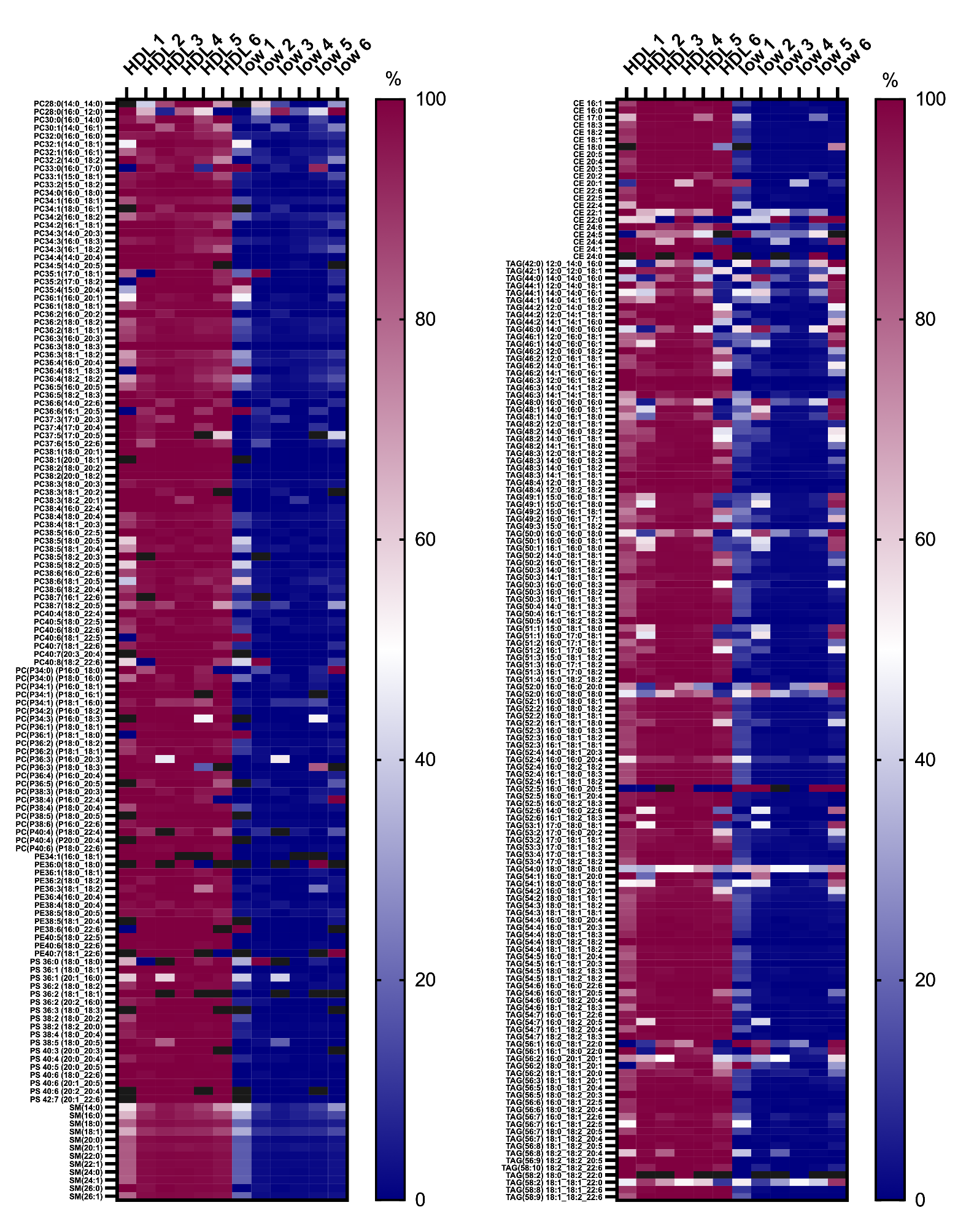


**Figure S2. Lipidomics comparing HDL (HDL) and the infranatant (low) following ultracentrifugation-based isolation with apoB-depleted plasma as starting material.** ApoB-depleted plasma was generated from 3 individual healthy male and female controls each as detailed in methods and adjusted with KBr to a density of d=1.21 g/mL. Then lipidomics analysis was carried out on the isolated HDL as well as the infranatant (low) as outlined in methods; results were volume adjusted and expressed as percent of each individual lipid species distributed over HDL or the infranatant. Black color indicates not detectable.

**Figure S3**. **Plasma levels of sex hormones during the course of gender affirming hormone therapy**. (A) and (C) Estradiol, (B) and (D) Testosterone. TW, transgender women; TM, transgender men; T0, baseline; T1, following 1 month of gonadal suppression treatment, when sex hormones were at castration level; T12, after 11 months of gender-affirming treatment. Kruskal-Wallis test was used to test for significance. Statistical significance is indicated as ****p<0.0001).

**Figure S4**. **Distribution of lipid groups separated by saturation level in transgender men and transgender women during the course of gender affirming hormone therapy**. Concentration of lipid species between groups separated by saturation level. Data are represented as log10(μg/ml). (A) phosphatidylcholines (PC), (B) alkenylphosphatidylcholines (PCP), (C) phosphatidylethanolamines (PE) and alkenylphosphatidylethanolamines (PEP), (D) phosphatidylserines (PS), (E) phosphatidylinositols (PI), (F) sphingomyelins (SM), (G) triacylglycerols (TAG), (H) cholesteryl esters (CE). TW, transgender women; TM, transgender men; T0, baseline; T1, following 1 month of gonadal suppression treatment, when sex hormones were at castration level; T12, after 11 months of gender-affirming treatment; db, double bond.

**Figure S5. Distribution of lipid species on HDL in transgender men and transgender women during the course of gender affirming hormone therapy.**  Distribution of the analyzed lipid species on (A) HDL surface and (B) HDL core. PC, phosphatidylcholines; SM, sphingomyelins; CE, cholesteryl esters; TAG, triacylglycerols; PCP, alkenylphosphatidylcholines; PS, phosphatidylserines; PE, phosphatidylethanolamines; PI, phosphatidylinositols; PEP, alkenylphosphatidylethanolamines; TW, transgender women; TM, transgender men; T0, baseline; T1, following 1 month of gonadal suppression treatment, when sex hormones were at castration level; T12, after 11 months of gender-affirming treatment. Zoom in for added clarity.

**Supplementary table 1.** Settings used for lipidomic analysis. m/z, mass to charge ratio, quantification ion -molecular mass of the quantification ion, ionization – ionization mode (N-negative, P- positive), internal standard – internal standard used for each lipid species. Cone V- cone voltage used, Ce – collision energy, ion/ion+adduct- the ion formed after ionization.

| **Name** | **m/z** | **quantification ion** | **ionization** | **Cone_V** | **Ce** | **ion/ion+adduct** | **Internal standard** |
| --- | --- | --- | --- | --- | --- | --- | --- |
| CE 12:0 | 586.6 | 369.3 | P | 45 | 30 | [M+NH4]+ | TAG(52:4) 16:0_20:4_16:0d9 |
| CE 14:0 | 610.6 | 369.3 | P | 45 | 30 | [M+NH4]+ | TAG(52:4) 16:0_20:4_16:0d9 |
| CE 15:0 | 628.6 | 369.3 | P | 45 | 30 | [M+NH4]+ | TAG(52:4) 16:0_20:4_16:0d9 |
| CE 16:1 | 640.6 | 369.3 | P | 45 | 30 | [M+NH4]+ | TAG(52:4) 16:0_20:4_16:0d9 |
| CE 16:0 | 642.6 | 369.3 | P | 45 | 30 | [M+NH4]+ | TAG(52:4) 16:0_20:4_16:0d9 |
| CE 17:0 | 656.6 | 369.3 | P | 45 | 30 | [M+NH4]+ | TAG(52:4) 16:0_20:4_16:0d9 |
| CE 18:3 | 664.6 | 369.3 | P | 45 | 30 | [M+NH4]+ | TAG(52:4) 16:0_20:4_16:0d9 |
| CE 18:2 | 666.6 | 369.3 | P | 45 | 30 | [M+NH4]+ | TAG(52:4) 16:0_20:4_16:0d9 |
| CE 18:1 | 668.6 | 369.3 | P | 45 | 30 | [M+NH4]+ | TAG(52:4) 16:0_20:4_16:0d9 |
| CE 18:0 | 670.7 | 369.3 | P | 45 | 30 | [M+NH4]+ | TAG(52:4) 16:0_20:4_16:0d9 |
| CE 20:5 | 688.6 | 369.3 | P | 45 | 30 | [M+NH4]+ | TAG(52:4) 16:0_20:4_16:0d9 |
| CE 20:4 | 690.6 | 369.3 | P | 45 | 30 | [M+NH4]+ | TAG(52:4) 16:0_20:4_16:0d9 |
| CE 20:3 | 692.6 | 369.3 | P | 45 | 30 | [M+NH4]+ | TAG(52:4) 16:0_20:4_16:0d9 |
| CE 20:2 | 694.7 | 369.3 | P | 45 | 30 | [M+NH4]+ | TAG(52:4) 16:0_20:4_16:0d9 |
| CE 20:1 | 696.7 | 369.3 | P | 45 | 30 | [M+NH4]+ | TAG(52:4) 16:0_20:4_16:0d9 |
| CE 22:6 | 714.6 | 369.3 | P | 45 | 30 | [M+NH4]+ | TAG(52:4) 16:0_20:4_16:0d9 |
| CE 22:5 | 716.6 | 369.3 | P | 45 | 30 | [M+NH4]+ | TAG(52:4) 16:0_20:4_16:0d9 |
| CE 22:4 | 718.7 | 369.3 | P | 45 | 30 | [M+NH4]+ | TAG(52:4) 16:0_20:4_16:0d9 |
| CE 22:1 | 724.7 | 369.3 | P | 45 | 30 | [M+NH4]+ | TAG(52:4) 16:0_20:4_16:0d9 |
| CE 22:0 | 726.7 | 369.3 | P | 45 | 30 | [M+NH4]+ | TAG(52:4) 16:0_20:4_16:0d9 |
| CE 24:6 | 742.7 | 369.3 | P | 45 | 30 | [M+NH4]+ | TAG(52:4) 16:0_20:4_16:0d9 |
| CE 24:5 | 744.7 | 369.3 | P | 45 | 30 | [M+NH4]+ | TAG(52:4) 16:0_20:4_16:0d9 |
| CE 24:4 | 746.7 | 369.3 | P | 45 | 30 | [M+NH4]+ | TAG(52:4) 16:0_20:4_16:0d9 |
| CE 24:1 | 752.7 | 369.3 | P | 45 | 30 | [M+NH4]+ | TAG(52:4) 16:0_20:4_16:0d9 |
| CE 24:0 | 754.7 | 369.3 | P | 45 | 30 | [M+NH4]+ | TAG(52:4) 16:0_20:4_16:0d9d |
| TAG(42:0) 12:0_14:0_16:0 | 740.7 | 523.5 | P | 30 | 14 | [M+NH4]+ | TAG(52:4) 16:0_20:4_16:0d9 |
| TAG(42:1) 12:0_12:0_18:1 | 738.7 | 439.4 | P | 30 | 14 | [M+NH4]+ | TAG(52:4) 16:0_20:4_16:0d9 |
| TAG(44:0) 14:0_14:0_16:0 | 768.7 | 495.4 | P | 30 | 14 | [M+NH4]+ | TAG(52:4) 16:0_20:4_16:0d9 |
| TAG(44:1) 12:0_14:0_18:1 | 766.7 | 549.5 | P | 30 | 14 | [M+NH4]+ | TAG(52:4) 16:0_20:4_16:0d9 |
| TAG(44:1) 14:0_14:0_16:1 | 766.7 | 495.4 | P | 30 | 14 | [M+NH4]+ | TAG(52:4) 16:0_20:4_16:0d9 |
| TAG(44:1) 14:0_14:1_16:0 | 766.7 | 493.4 | P | 30 | 14 | [M+NH4]+ | TAG(52:4) 16:0_20:4_16:0d9 |
| TAG(44:2) 12:0_14:0_18:2 | 764.7 | 467.4 | P | 30 | 14 | [M+NH4]+ | TAG(52:4) 16:0_20:4_16:0d9 |
| TAG(44:2) 12:0_14:1_18:1 | 764.7 | 465.4 | P | 30 | 14 | [M+NH4]+ | TAG(52:4) 16:0_20:4_16:0d9 |
| TAG(44:2) 14:1_14:1_16:0 | 764.7 | 491.4 | P | 30 | 14 | [M+NH4]+ | TAG(52:4) 16:0_20:4_16:0d9 |
| TAG(46:0) 14:0_16:0_16:0 | 796.7 | 523.4 | P | 30 | 14 | [M+NH4]+ | TAG(52:4) 16:0_20:4_16:0d9 |
| TAG(46:1) 12:0_16:0_18:1 | 794.7 | 495.4 | P | 30 | 14 | [M+NH4]+ | TAG(52:4) 16:0_20:4_16:0d9 |
| TAG(46:1) 14:0_16:0_16:1 | 794.7 | 549.5 | P | 30 | 14 | [M+NH4]+ | TAG(52:4) 16:0_20:4_16:0d9 |
| TAG(46:2) 12:0_16:0_18:2 | 792.7 | 575.5 | P | 30 | 14 | [M+NH4]+ | TAG(52:4) 16:0_20:4_16:0d9 |
| TAG(46:2) 12:0_16:1_18:1 | 792.7 | 493.4 | P | 30 | 14 | [M+NH4]+ | TAG(52:4) 16:0_20:4_16:0d9 |
| TAG(46:2) 14:0_16:1_16:1 | 792.7 | 547.5 | P | 30 | 14 | [M+NH4]+ | TAG(52:4) 16:0_20:4_16:0d9 |
| TAG(46:2) 14:1_16:0_16:1 | 792.7 | 549.5 | P | 30 | 14 | [M+NH4]+ | TAG(52:4) 16:0_20:4_16:0d9 |
| TAG(46:3) 12:0_16:1_18:2 | 790.7 | 573.5 | P | 30 | 14 | [M+NH4]+ | TAG(52:4) 16:0_20:4_16:0d9 |
| TAG(46:3) 14:0_14:1_18:2 | 790.7 | 493.4 | P | 30 | 14 | [M+NH4]+ | TAG(52:4) 16:0_20:4_16:0d9 |
| TAG(46:3) 14:1_14:1_18:1 | 790.7 | 491.4 | P | 30 | 14 | [M+NH4]+ | TAG(52:4) 16:0_20:4_16:0d9 |
| TAG(48:0) 16:0_16:0_16:0 | 824.8 | 551.5 | P | 30 | 14 | [M+NH4]+ | TAG(52:4) 16:0_20:4_16:0d9 |
| TAG(48:1) 14:0_16:0_18:1 | 822.8 | 549.5 | P | 30 | 14 | [M+NH4]+ | TAG(52:4) 16:0_20:4_16:0d9 |
| TAG(48:1) 14:0_16:1_18:0 | 822.7 | 551.4 | P | 30 | 14 | [M+NH4]+ | TAG(52:4) 16:0_20:4_16:0d9 |
| TAG(48:2) 12:0_18:1_18:1 | 820.7 | 603.5 | P | 30 | 14 | [M+NH4]+ | TAG(52:4) 16:0_20:4_16:0d9 |
| TAG(48:2) 14:0_16:0_18:2 | 820.7 | 547.4 | P | 30 | 14 | [M+NH4]+ | TAG(52:4) 16:0_20:4_16:0d9 |
| TAG(48:2) 14:0_16:1_18:1 | 820.7 | 575.5 | P | 30 | 14 | [M+NH4]+ | TAG(52:4) 16:0_20:4_16:0d9 |
| TAG(48:2) 14:1_16:1_18:0 | 820.7 | 577.6 | P | 30 | 14 | [M+NH4]+ | TAG(52:4) 16:0_20:4_16:0d9 |
| TAG(48:3) 12:0_18:1_18:2 | 818.7 | 601.5 | P | 30 | 14 | [M+NH4]+ | TAG(52:4) 16:0_20:4_16:0d9 |
| TAG(48:3) 14:0_16:0_18:3 | 818.7 | 523.4 | P | 30 | 14 | [M+NH4]+ | TAG(52:4) 16:0_20:4_16:0d9 |
| TAG(48:3) 14:0_16:1_18:2 | 818.7 | 521.5 | P | 30 | 14 | [M+NH4]+ | TAG(52:4) 16:0_20:4_16:0d9 |
| TAG(48:3) 14:1_16:1_18:1 | 818.7 | 519.4 | P | 30 | 14 | [M+NH4]+ | TAG(52:4) 16:0_20:4_16:0d9 |
| TAG(48:4) 12:0_18:1_18:3 | 816.7 | 521.4 | P | 30 | 14 | [M+NH4]+ | TAG(52:4) 16:0_20:4_16:0d9 |
| TAG(48:4) 12:0_18:2_18:2 | 816.7 | 519.4 | P | 30 | 14 | [M+NH4]+ | TAG(52:4) 16:0_20:4_16:0d9 |
| TAG(49:1) 15:0_16:0_18:1 | 836.8 | 537.5 | P | 30 | 14 | [M+NH4]+ | TAG(52:4) 16:0_20:4_16:0d9 |
| TAG(49:1) 15:0_16:1_18:0 | 836.8 | 577.5 | P | 30 | 14 | [M+NH4]+ | TAG(52:4) 16:0_20:4_16:0d9 |
| TAG(49:2) 15:0_16:1_18:1 | 834.8 | 535.5 | P | 30 | 14 | [M+NH4]+ | TAG(52:4) 16:0_20:4_16:0d9 |
| TAG(49:2) 16:0_16:1_17:1 | 834.8 | 561.5 | P | 30 | 14 | [M+NH4]+ | TAG(52:4) 16:0_20:4_16:0d9 |
| TAG(49:3) 15:0_16:1_18:2 | 832.7 | 535.5 | P | 30 | 14 | [M+NH4]+ | TAG(52:4) 16:0_20:4_16:0d9 |
| TAG(50:0) 16:0_16:0_18:0 | 852.8 | 579.5 | P | 30 | 14 | [M+NH4]+ | TAG(52:4) 16:0_20:4_16:0d9 |
| TAG(50:1) 16:0_16:0_18:1 | 850.8 | 551.5 | P | 30 | 14 | [M+NH4]+ | TAG(52:4) 16:0_20:4_16:0d9 |
| TAG(50:1) 16:1_16:0_18:0 | 850.8 | 549.5 | P | 30 | 14 | [M+NH4]+ | TAG(52:4) 16:0_20:4_16:0d9 |
| TAG(50:2) 14:0_18:1_18:1 | 848.8 | 603.5 | P | 30 | 14 | [M+NH4]+ | TAG(52:4) 16:0_20:4_16:0d9 |
| TAG(50:2) 16:0_16:1_18:1 | 848.8 | 577.5 | P | 30 | 14 | [M+NH4]+ | TAG(52:4) 16:0_20:4_16:0d9 |
| TAG(50:3) 14:0_18:1_18:2 | 846.8 | 547.5 | P | 30 | 14 | [M+NH4]+ | TAG(52:4) 16:0_20:4_16:0d9 |
| TAG(50:3) 14:1_18:1_18:1 | 846.8 | 603.6 | P | 30 | 14 | [M+NH4]+ | TAG(52:4) 16:0_20:4_16:0d9 |
| TAG(50:3) 16:0_16:0_18:3 | 846.9 | 551.5 | P | 30 | 14 | [M+NH4]+ | TAG(52:4) 16:0_20:4_16:0d9 |
| TAG(50:3) 16:0_16:1_18:2 | 846.8 | 549.5 | P | 30 | 14 | [M+NH4]+ | TAG(52:4) 16:0_20:4_16:0d9 |
| TAG(50:3) 16:1_16:1_18:1 | 846.8 | 547.5 | P | 30 | 14 | [M+NH4]+ | TAG(52:4) 16:0_20:4_16:0d9 |
| TAG(50:4) 14:0_18:1_18:3 | 844.6 | 549.3 | P | 30 | 14 | [M+NH4]+ | TAG(52:4) 16:0_20:4_16:0d9 |
| TAG(50:4) 16:1_16:1_18:2 | 844.7 | 573.4 | P | 30 | 14 | [M+NH4]+ | TAG(52:4) 16:0_20:4_16:0d9 |
| TAG(50:5) 14:0_18:2_18:3 | 842.7 | 597.5 | P | 30 | 14 | [M+NH4]+ | TAG(52:4) 16:0_20:4_16:0d9 |
| TAG(51:1) 15:0_18:1_18:0 | 864.8 | 565.5 | P | 30 | 14 | [M+NH4]+ | TAG(52:4) 16:0_20:4_16:0d9 |
| TAG(51:1) 16:0_17:0_18:1 | 864.8 | 591.5 | P | 30 | 14 | [M+NH4]+ | TAG(52:4) 16:0_20:4_16:0d9 |
| TAG(51:2) 16:0_17:1_18:1 | 862.8 | 577.5 | P | 30 | 14 | [M+NH4]+ | TAG(52:4) 16:0_20:4_16:0d9 |
| TAG(51:2) 16:1_17:0_18:1 | 862.8 | 575.5 | P | 30 | 14 | [M+NH4]+ | TAG(52:4) 16:0_20:4_16:0d9 |
| TAG(51:3) 15:0_18:1_18:2 | 860.8 | 601.5 | P | 30 | 14 | [M+NH4]+ | TAG(52:4) 16:0_20:4_16:0d9 |
| TAG(51:3) 16:0_17:1_18:2 | 860.8 | 575.5 | P | 30 | 14 | [M+NH4]+ | TAG(52:4) 16:0_20:4_16:0d9 |
| TAG(51:3) 16:1_17:0_18:2 | 860.8 | 589.5 | P | 30 | 14 | [M+NH4]+ | TAG(52:4) 16:0_20:4_16:0d9 |
| TAG(51:4) 15:0_18:2_18:2 | 858.8 | 561.5 | P | 30 | 14 | [M+NH4]+ | TAG(52:4) 16:0_20:4_16:0d9 |
| TAG(52:0) 16:0_16:0_20:0 | 880.8 | 551.5 | P | 30 | 14 | [M+NH4]+ | TAG(52:4) 16:0_20:4_16:0d9 |
| TAG(52:0) 16:0_18:0_18:0 | 880.8 | 579.5 | P | 30 | 14 | [M+NH4]+ | TAG(52:4) 16:0_20:4_16:0d9 |
| TAG(52:1) 16:0_18:0_18:1 | 878.8 | 579.5 | P | 30 | 14 | [M+NH4]+ | TAG(52:4) 16:0_20:4_16:0d9 |
| TAG(52:2) 16:0_18:0_18:2 | 876.8 | 579.5 | P | 30 | 14 | [M+NH4]+ | TAG(52:4) 16:0_20:4_16:0d9 |
| TAG(52:2) 16:0_18:1_18:1 | 876.8 | 603.5 | P | 30 | 14 | [M+NH4]+ | TAG(52:4) 16:0_20:4_16:0d9 |
| TAG(52:2) 16:1_18:1_18:0 | 876.8 | 575.5 | P | 30 | 14 | [M+NH4]+ | TAG(52:4) 16:0_20:4_16:0d9 |
| TAG(52:3) 16:0_18:0_18:3 | 874.8 | 579.5 | P | 30 | 14 | [M+NH4]+ | TAG(52:4) 16:0_20:4_16:0d9 |
| TAG(52:3) 16:0_18:1_18:2 | 874.8 | 577.5 | P | 30 | 14 | [M+NH4]+ | TAG(52:4) 16:0_20:4_16:0d9 |
| TAG(52:3) 16:1_18:1_18:1 | 874.8 | 575.5 | P | 30 | 14 | [M+NH4]+ | TAG(52:4) 16:0_20:4_16:0d9 |
| TAG(52:4) 14:0_18:1_20:3 | 872.8 | 549.5 | P | 30 | 14 | [M+NH4]+ | TAG(52:4) 16:0_20:4_16:0d9 |
| TAG(52:4) 16:0_16:0_20:4 | 872.8 | 551.5 | P | 30 | 14 | [M+NH4]+ | TAG(52:4) 16:0_20:4_16:0d9 |
| TAG(52:4) 16:0_18:2_18:2 | 872.8 | 599.5 | P | 30 | 14 | [M+NH4]+ | TAG(52:4) 16:0_20:4_16:0d9 |
| TAG(52:4) 16:1_18:0_18:3 | 872.8 | 577.5 | P | 30 | 14 | [M+NH4]+ | TAG(52:4) 16:0_20:4_16:0d9 |
| TAG(52:4) 16:1_18:1_18:2 | 872.8 | 601.5 | P | 30 | 14 | [M+NH4]+ | TAG(52:4) 16:0_20:4_16:0d9 |
| TAG(52:5) 16:0_16:1_20:4 | 870.8 | 549.5 | P | 30 | 14 | [M+NH4]+ | TAG(52:4) 16:0_20:4_16:0d9 |
| TAG(52:5) 16:0_18:2_18:3 | 870.8 | 597.5 | P | 30 | 14 | [M+NH4]+ | TAG(52:4) 16:0_20:4_16:0d9 |
| TAG(52:6) 14:0_16:0_22:6 | 868.8 | 595.5 | P | 30 | 14 | [M+NH4]+ | TAG(52:4) 16:0_20:4_16:0d9 |
| TAG(52:6) 16:1_18:2_18:3 | 868.7 | 571.4 | P | 30 | 14 | [M+NH4]+ | TAG(52:4) 16:0_20:4_16:0d9 |
| TAG(53:1) 17:0_18:0_18:1 | 892.8 | 591.5 | P | 30 | 14 | [M+NH4]+ | TAG(52:4) 16:0_20:4_16:0d9 |
| TAG(53:2) 17:0_16:0_20:2 | 890.8 | 617.5 | P | 30 | 14 | [M+NH4]+ | TAG(52:4) 16:0_20:4_16:0d9 |
| TAG(53:2) 17:0_18:1_18:1 | 890.8 | 591.5 | P | 30 | 14 | [M+NH4]+ | TAG(52:4) 16:0_20:4_16:0d9 |
| TAG(53:3) 17:0_18:1_18:2 | 888.8 | 591.5 | P | 30 | 14 | [M+NH4]+ | TAG(52:4) 16:0_20:4_16:0d9 |
| TAG(53:4) 17:0_18:1_18:3 | 886.8 | 587.5 | P | 30 | 14 | [M+NH4]+ | TAG(52:4) 16:0_20:4_16:0d9 |
| TAG(53:4) 17:0_18:2_18:2 | 886.8 | 589.5 | P | 30 | 14 | [M+NH4]+ | TAG(52:4) 16:0_20:4_16:0d9 |
| TAG(54:0) 18:0_18:0_18:0 | 908.9 | 607.7 | P | 30 | 14 | [M+NH4]+ | TAG(52:4) 16:0_20:4_16:0d9 |
| TAG(54:1) 16:0_18:1_20:0 | 906.8 | 577.5 | P | 30 | 14 | [M+NH4]+ | TAG(52:4) 16:0_20:4_16:0d9 |
| TAG(54:1) 18:0_18:0_18:1 | 906.8 | 607.6 | P | 30 | 14 | [M+NH4]+ | TAG(52:4) 16:0_20:4_16:0d9 |
| TAG(54:2) 16:0_18:1_20:1 | 904.8 | 577.5 | P | 30 | 14 | [M+NH4]+ | TAG(52:4) 16:0_20:4_16:0d9 |
| TAG(54:2) 18:0_18:1_18:1 | 904.8 | 603.6 | P | 30 | 14 | [M+NH4]+ | TAG(52:4) 16:0_20:4_16:0d9 |
| TAG(54:3) 18:0_18:1_18:2 | 902.8 | 605.5 | P | 30 | 14 | [M+NH4]+ | TAG(52:4) 16:0_20:4_16:0d9 |
| TAG(54:3) 18:1_18:1_18:1 | 902.8 | 603.6 | P | 30 | 14 | [M+NH4]+ | TAG(52:4) 16:0_20:4_16:0d9 |
| TAG(54:4) 16:0_18:0_20:4 | 900.8 | 627.5 | P | 30 | 14 | [M+NH4]+ | TAG(52:4) 16:0_20:4_16:0d9 |
| TAG(54:4) 16:0_18:1_20:3 | 900.8 | 577.5 | P | 30 | 14 | [M+NH4]+ | TAG(52:4) 16:0_20:4_16:0d9 |
| TAG(54:4) 18:0_18:1_18:3 | 900.8 | 605.5 | P | 30 | 14 | [M+NH4]+ | TAG(52:4) 16:0_20:4_16:0d9 |
| TAG(54:4) 18:0_18:2_18:2 | 900.8 | 599.5 | P | 30 | 14 | [M+NH4]+ | TAG(52:4) 16:0_20:4_16:0d9 |
| TAG(54:4) 18:1_18:1_18:2 | 900.8 | 601.5 | P | 30 | 14 | [M+NH4]+ | TAG(52:4) 16:0_20:4_16:0d9 |
| TAG(54:5) 16:0_18:1_20:4 | 898.8 | 577.5 | P | 30 | 14 | [M+NH4]+ | TAG(52:4) 16:0_20:4_16:0d9 |
| TAG(54:5) 16:1_18:1_20:3 | 898.8 | 575.5 | P | 30 | 14 | [M+NH4]+ | TAG(52:4) 16:0_20:4_16:0d9 |
| TAG(54:5) 18:0_18:2_18:3 | 898.8 | 603.5 | P | 30 | 14 | [M+NH4]+ | TAG(52:4) 16:0_20:4_16:0d9 |
| TAG(54:5) 18:1_18:2_18:2 | 898.8 | 599.5 | P | 30 | 14 | [M+NH4]+ | TAG(52:4) 16:0_20:4_16:0d9 |
| TAG(54:6) 16:0_16:0_22:6 | 896.8 | 551.5 | P | 30 | 14 | [M+NH4]+ | TAG(52:4) 16:0_20:4_16:0d9 |
| TAG(54:6) 16:0_18:1_20:5 | 896.8 | 577.5 | P | 30 | 14 | [M+NH4]+ | TAG(52:4) 16:0_20:4_16:0d9 |
| TAG(54:6) 16:0_18:2_20:4 | 896.8 | 575.5 | P | 30 | 14 | [M+NH4]+ | TAG(52:4) 16:0_20:4_16:0d9 |
| TAG(54:6) 18:1_18:2_18:3 | 896.7 | 601.5 | P | 30 | 14 | [M+NH4]+ | TAG(52:4) 16:0_20:4_16:0d9 |
| TAG(54:7) 16:0_16:1_22:6 | 894.8 | 549.5 | P | 30 | 14 | [M+NH4]+ | TAG(52:4) 16:0_20:4_16:0d9 |
| TAG(54:7) 16:0_18:2_20:5 | 894.8 | 575.5 | P | 30 | 14 | [M+NH4]+ | TAG(52:4) 16:0_20:4_16:0d9 |
| TAG(54:7) 16:1_18:2_20:4 | 894.8 | 573.5 | P | 30 | 14 | [M+NH4]+ | TAG(52:4) 16:0_20:4_16:0d9 |
| TAG(54:7) 18:2_18:2_18:3 | 894.8 | 599.5 | P | 30 | 14 | [M+NH4]+ | TAG(52:4) 16:0_20:4_16:0d9 |
| TAG(56:1) 16:0_18:1_22:0 | 934.9 | 661.6 | P | 30 | 14 | [M+NH4]+ | TAG(52:4) 16:0_20:4_16:0d9 |
| TAG(56:1) 16:1_18:0_22:0 | 934.9 | 633.6 | P | 30 | 14 | [M+NH4]+ | TAG(52:4) 16:0_20:4_16:0d9 |
| TAG(56:2) 16:0_20:1_20:1 | 932.9 | 659.6 | P | 30 | 14 | [M+NH4]+ | TAG(52:4) 16:0_20:4_16:0d9 |
| TAG(56:2) 18:0_18:1_20:1 | 932.9 | 631.6 | P | 30 | 14 | [M+NH4]+ | TAG(52:4) 16:0_20:4_16:0d9 |
| TAG(56:2) 18:1_18:1_20:0 | 932.9 | 603.6 | P | 30 | 14 | [M+NH4]+ | TAG(52:4) 16:0_20:4_16:0d9 |
| TAG(56:3) 18:1_18:1_20:1 | 930.8 | 603.5 | P | 30 | 14 | [M+NH4]+ | TAG(52:4) 16:0_20:4_16:0d9 |
| TAG(56:5) 18:0_18:1_20:4 | 926.8 | 605.5 | P | 30 | 14 | [M+NH4]+ | TAG(52:4) 16:0_20:4_16:0d9 |
| TAG(56:5) 18:0_18:2_20:3 | 926.8 | 603.5 | P | 30 | 14 | [M+NH4]+ | TAG(52:4) 16:0_20:4_16:0d9 |
| TAG(56:6) 16:0_18:1_22:5 | 924.8 | 577.5 | P | 30 | 14 | [M+NH4]+ | TAG(52:4) 16:0_20:4_16:0d9 |
| TAG(56:6) 18:0_18:2_20:4 | 924.8 | 603.5 | P | 30 | 14 | [M+NH4]+ | TAG(52:4) 16:0_20:4_16:0d9 |
| TAG(56:7) 16:0_18:1_22:6 | 922.8 | 649.5 | P | 30 | 14 | [M+NH4]+ | TAG(52:4) 16:0_20:4_16:0d9 |
| TAG(56:7) 16:1_18:1_22:5 | 922.8 | 575.5 | P | 30 | 14 | [M+NH4]+ | TAG(52:4) 16:0_20:4_16:0d9 |
| TAG(56:7) 18:0_18:2_20:5 | 922.8 | 603.5 | P | 30 | 14 | [M+NH4]+ | TAG(52:4) 16:0_20:4_16:0d9 |
| TAG(56:7) 18:1_18:2_20:4 | 922.8 | 601.5 | P | 30 | 14 | [M+NH4]+ | TAG(52:4) 16:0_20:4_16:0d9 |
| TAG(56:8) 18:1_18:2_20:5 | 920.8 | 601.5 | P | 30 | 14 | [M+NH4]+ | TAG(52:4) 16:0_20:4_16:0d9 |
| TAG(56:8) 18:2_18:2_20:4 | 920.8 | 599.5 | P | 30 | 14 | [M+NH4]+ | TAG(52:4) 16:0_20:4_16:0d9 |
| TAG(56:9) 18:2_18:2_20:5 | 918.8 | 599.5 | P | 30 | 14 | [M+NH4]+ | TAG(52:4) 16:0_20:4_16:0d9 |
| TAG(58:10) 18:2_18:2_22:6 | 944.8 | 599.5 | P | 30 | 14 | [M+NH4]+ | TAG(52:4) 16:0_20:4_16:0d9 |
| TAG(58:2) 18:0_18:2_22:0 | 960.9 | 659.6 | P | 30 | 14 | [M+NH4]+ | TAG(52:4) 16:0_20:4_16:0d9 |
| TAG(58:2) 18:1_18:1_22:0 | 960.9 | 661.6 | P | 30 | 14 | [M+NH4]+ | TAG(52:4) 16:0_20:4_16:0d9 |
| TAG(58:8) 18:1_18:1_22:6 | 948.8 | 603.5 | P | 30 | 14 | [M+NH4]+ | TAG(52:4) 16:0_20:4_16:0d9 |
| TAG(58:9) 18:1_18:2_22:6 | 946.8 | 601.5 | P | 30 | 14 | [M+NH4]+ | TAG(52:4) 16:0_20:4_16:0d9 |
| PC26:0(12:0_14:0) | 694.5 | 227.2 | N | 30 | 24 | [M-COO]- | PC36:4 (16:0d9-20:4) |
| PC28:0(14:0_14:0) | 722.5 | 227.2 | N | 30 | 24 | [M-COO]- | PC36:4 (16:0d9-20:4) |
| PC28:0(16:0_12:0) | 722.5 | 255.2 | N | 30 | 24 | [M-COO]- | PC36:4 (16:0d9-20:4) |
| PC28:1(12:0_16:1) | 720.5 | 253.2 | N | 30 | 24 | [M-COO]- | PC36:4 (16:0d9-20:4) |
| PC30:0(16:0_14:0) | 750.5 | 227.2 | N | 30 | 24 | [M-COO]- | PC36:4 (16:0d9-20:4) |
| PC30:0(18:0_12:0) | 750.5 | 283.3 | N | 30 | 24 | [M-COO]- | PC36:4 (16:0d9-20:4) |
| PC30:1(12:0_18:1) | 748.5 | 225.2 | N | 30 | 24 | [M-COO]- | PC36:4 (16:0d9-20:4) |
| PC30:1(14:0_16:1) | 748.5 | 253.2 | N | 30 | 24 | [M-COO]- | PC36:4 (16:0d9-20:4) |
| PC32:0(14:0_18:0) | 778.5 | 227.2 | N | 30 | 24 | [M-COO]- | PC36:4 (16:0d9-20:4) |
| PC32:0(16:0_16:0) | 778.5 | 255.2 | N | 30 | 24 | [M-COO]- | PC36:4 (16:0d9-20:4) |
| PC32:1(14:0_18:1) | 776.5 | 281.2 | N | 30 | 24 | [M-COO]- | PC36:4 (16:0d9-20:4) |
| PC32:1(16:0_16:1) | 776.5 | 253.2 | N | 30 | 24 | [M-COO]- | PC36:4 (16:0d9-20:4) |
| PC32:2(14:0_18:2) | 774.5 | 279.2 | N | 30 | 24 | [M-COO]- | PC36:4 (16:0d9-20:4) |
| PC32:2(16:1_16:1) | 774.5 | 253.2 | N | 30 | 24 | [M-COO]- | PC36:4 (16:0d9-20:4) |
| PC32:2(18:1_14:1) | 774.5 | 225.2 | N | 30 | 24 | [M-COO]- | PC36:4 (16:0d9-20:4) |
| PC32:3(14:0_18:3) | 772.5 | 277.2 | N | 30 | 24 | [M-COO]- | PC36:4 (16:0d9-20:4) |
| PC33:0(16:0_17:0) | 792.6 | 269.2 | N | 30 | 24 | [M-COO]- | PC36:4 (16:0d9-20:4) |
| PC33:1(15:0_18:1) | 790.6 | 241.2 | N | 30 | 24 | [M-COO]- | PC36:4 (16:0d9-20:4) |
| PC33:2(15:0_18:2) | 788.5 | 241.2 | N | 30 | 24 | [M-COO]- | PC36:4 (16:0d9-20:4) |
| PC33:3(15:0_18:3) | 786.5 | 241.2 | N | 30 | 24 | [M-COO]- | PC36:4 (16:0d9-20:4) |
| PC34:0(16:0_18:0) | 806.6 | 283.3 | N | 30 | 24 | [M-COO]- | PC36:4 (16:0d9-20:4) |
| PC34:1(16:0_18:1) | 804.6 | 281.2 | N | 30 | 24 | [M-COO]- | PC36:4 (16:0d9-20:4) |
| PC34:1(18:0_16:1) | 804.6 | 253.2 | N | 30 | 24 | [M-COO]- | PC36:4 (16:0d9-20:4) |
| PC34:2(16:0_18:2) | 802.5 | 279.2 | N | 30 | 24 | [M-COO]- | PC36:4 (16:0d9-20:4) |
| PC34:2(16:1_18:1) | 802.5 | 281.2 | N | 30 | 24 | [M-COO]- | PC36:4 (16:0d9-20:4) |
| PC34:3(14:0_20:3) | 800.5 | 305.2 | N | 30 | 24 | [M-COO]- | PC36:4 (16:0d9-20:4) |
| PC34:3(16:0_18:3) | 800.5 | 277.2 | N | 30 | 24 | [M-COO]- | PC36:4 (16:0d9-20:4) |
| PC34:3(16:1_18:2) | 800.5 | 279.2 | N | 30 | 24 | [M-COO]- | PC36:4 (16:0d9-20:4) |
| PC34:4(14:0_20:4) | 798.5 | 303.2 | N | 30 | 24 | [M-COO]- | PC36:4 (16:0d9-20:4) |
| PC34:4(16:1_18:3) | 798.5 | 277.2 | N | 30 | 24 | [M-COO]- | PC36:4 (16:0d9-20:4) |
| PC34:5(14:0_20:5) | 796.5 | 301.2 | N | 30 | 24 | [M-COO]- | PC36:4 (16:0d9-20:4) |
| PC35:1(17:0_18:1) | 818.6 | 269.2 | N | 30 | 24 | [M-COO]- | PC36:4 (16:0d9-20:4) |
| PC35:2(17:0_18:2) | 816.6 | 269.2 | N | 30 | 24 | [M-COO]- | PC36:4 (16:0d9-20:4) |
| PC35:3(15:0_20:3) | 814.6 | 241.2 | N | 30 | 24 | [M-COO]- | PC36:4 (16:0d9-20:4) |
| PC35:3(17:1_18:2) | 814.6 | 267.2 | N | 30 | 24 | [M-COO]- | PC36:4 (16:0d9-20:4) |
| PC35:4(15:0_20:4) | 812.5 | 241.2 | N | 30 | 24 | [M-COO]- | PC36:4 (16:0d9-20:4) |
| PC35:5(15:0_20:5) | 810.5 | 241.2 | N | 30 | 24 | [M-COO]- | PC36:4 (16:0d9-20:4) |
| PC36:0(18:0_18:0) | 834.6 | 283.3 | N | 30 | 24 | [M-COO]- | PC36:4 (16:0d9-20:4) |
| PC36:1(16:0_20:1) | 832.6 | 309.3 | N | 30 | 24 | [M-COO]- | PC36:4 (16:0d9-20:4) |
| PC36:1(18:0_18:1) | 832.6 | 281.2 | N | 30 | 24 | [M-COO]- | PC36:4 (16:0d9-20:4) |
| PC36:2(16:0_20:2) | 830.6 | 307.3 | N | 30 | 24 | [M-COO]- | PC36:4 (16:0d9-20:4) |
| PC36:2(18:0_18:2) | 830.6 | 279.2 | N | 30 | 24 | [M-COO]- | PC36:4 (16:0d9-20:4) |
| PC36:2(18:1_18:1) | 830.6 | 281.2 | N | 30 | 24 | [M-COO]- | PC36:4 (16:0d9-20:4) |
| PC36:3(16:0_20:3) | 828.6 | 305.2 | N | 30 | 24 | [M-COO]- | PC36:4 (16:0d9-20:4) |
| PC36:3(18:0_18:3) | 828.6 | 277.2 | N | 30 | 24 | [M-COO]- | PC36:4 (16:0d9-20:4) |
| PC36:3(18:1_18:2) | 828.6 | 279.2 | N | 30 | 24 | [M-COO]- | PC36:4 (16:0d9-20:4) |
| PC36:4(16:0_20:4) | 826.5 | 303.2 | N | 30 | 24 | [M-COO]- | PC36:4 (16:0d9-20:4) |
| PC36:4(18:1_18:3) | 826.5 | 277.2 | N | 30 | 24 | [M-COO]- | PC36:4 (16:0d9-20:4) |
| PC36:4(18:2_18:2) | 826.5 | 279.2 | N | 30 | 24 | [M-COO]- | PC36:4 (16:0d9-20:4) |
| PC36:5(16:0_20:5) | 824.5 | 301.2 | N | 30 | 24 | [M-COO]- | PC36:4 (16:0d9-20:4) |
| PC36:5(18:2_18:3) | 824.5 | 277.2 | N | 30 | 24 | [M-COO]- | PC36:4 (16:0d9-20:4) |
| PC36:6(14:0_22:6) | 822.5 | 327.2 | N | 30 | 24 | [M-COO]- | PC36:4 (16:0d9-20:4) |
| PC36:6(16:1_20:5) | 822.5 | 301.2 | N | 30 | 24 | [M-COO]- | PC36:4 (16:0d9-20:4) |
| PC36:6(18:3_18:3) | 822.5 | 277.2 | N | 30 | 24 | [M-COO]- | PC36:4 (16:0d9-20:4) |
| PC37:3(17:0_20:3) | 842.6 | 269.2 | N | 30 | 24 | [M-COO]- | PC36:4 (16:0d9-20:4) |
| PC37:4(17:0_20:4) | 840.6 | 269.2 | N | 30 | 24 | [M-COO]- | PC36:4 (16:0d9-20:4) |
| PC37:5(17:0_20:5) | 838.6 | 269.2 | N | 30 | 24 | [M-COO]- | PC36:4 (16:0d9-20:4) |
| PC37:5(17:1_20:4) | 838.6 | 267.2 | N | 30 | 24 | [M-COO]- | PC36:4 (16:0d9-20:4) |
| PC37:6(15:0_22:6) | 836.5 | 241.2 | N | 30 | 24 | [M-COO]- | PC36:4 (16:0d9-20:4) |
| PC38:1(18:0_20:1) | 860.6 | 309.3 | N | 30 | 24 | [M-COO]- | PC36:4 (16:0d9-20:4) |
| PC38:1(20:0_18:1) | 860.6 | 281.3 | N | 30 | 24 | [M-COO]- | PC36:4 (16:0d9-20:4) |
| PC38:2(18:0_20:2) | 858.6 | 307.3 | N | 30 | 24 | [M-COO]- | PC36:4 (16:0d9-20:4) |
| PC38:2(18:1_20:1) | 858.6 | 309.3 | N | 30 | 24 | [M-COO]- | PC36:4 (16:0d9-20:4) |
| PC38:2(20:0_18:2) | 858.6 | 279.2 | N | 30 | 24 | [M-COO]- | PC36:4 (16:0d9-20:4) |
| PC38:3(18:0_20:3) | 856.6 | 305.2 | N | 30 | 24 | [M-COO]- | PC36:4 (16:0d9-20:4) |
| PC38:3(18:1_20:2) | 856.6 | 307.3 | N | 30 | 24 | [M-COO]- | PC36:4 (16:0d9-20:4) |
| PC38:3(18:2_20:1) | 856.6 | 309.3 | N | 30 | 24 | [M-COO]- | PC36:4 (16:0d9-20:4) |
| PC38:4(16:0_22:4) | 854.6 | 331.3 | N | 30 | 24 | [M-COO]- | PC36:4 (16:0d9-20:4) |
| PC38:4(18:0_20:4) | 854.6 | 303.2 | N | 30 | 24 | [M-COO]- | PC36:4 (16:0d9-20:4) |
| PC38:4(18:1_20:3) | 854.6 | 305.2 | N | 30 | 24 | [M-COO]- | PC36:4 (16:0d9-20:4) |
| PC38:4(18:2_20:2) | 854.6 | 307.3 | N | 30 | 24 | [M-COO]- | PC36:4 (16:0d9-20:4) |
| PC38:5(16:0_22:5) | 852.6 | 329.2 | N | 30 | 24 | [M-COO]- | PC36:4 (16:0d9-20:4) |
| PC38:5(18:0_20:5) | 852.6 | 301.2 | N | 30 | 24 | [M-COO]- | PC36:4 (16:0d9-20:4) |
| PC38:5(18:1_20:4) | 852.6 | 303.2 | N | 30 | 24 | [M-COO]- | PC36:4 (16:0d9-20:4) |
| PC38:5(18:2_20:3) | 852.6 | 305.2 | N | 30 | 24 | [M-COO]- | PC36:4 (16:0d9-20:4) |
| PC38:5(18:2_20:5) | 852.6 | 301.2 | N | 30 | 24 | [M-COO]- | PC36:4 (16:0d9-20:4) |
| PC38:6(16:0_22:6) | 850.5 | 327.2 | N | 30 | 24 | [M-COO]- | PC36:4 (16:0d9-20:4) |
| PC38:6(18:1_20:5) | 850.5 | 301.2 | N | 30 | 24 | [M-COO]- | PC36:4 (16:0d9-20:4) |
| PC38:6(18:2_20:4) | 850.5 | 303.2 | N | 30 | 24 | [M-COO]- | PC36:4 (16:0d9-20:4) |
| PC38:7(16:1_22:6) | 848.5 | 327.2 | N | 30 | 24 | [M-COO]- | PC36:4 (16:0d9-20:4) |
| PC38:7(18:2_20:5) | 848.5 | 301.2 | N | 30 | 24 | [M-COO]- | PC36:4 (16:0d9-20:4) |
| PC38:7(18:3_20:4) | 848.5 | 303.2 | N | 30 | 24 | [M-COO]- | PC36:4 (16:0d9-20:4) |
| PC40:2(20:0_20:2) | 886.6 | 307.3 | N | 30 | 24 | [M-COO]- | PC36:4 (16:0d9-20:4) |
| PC40:3(18:2_22:1) | 884.6 | 337.3 | N | 30 | 24 | [M-COO]- | PC36:4 (16:0d9-20:4) |
| PC40:3(20:0_20:3) | 884.6 | 305.2 | N | 30 | 24 | [M-COO]- | PC36:4 (16:0d9-20:4) |
| PC40:4(18:0_22:4) | 882.6 | 331.3 | N | 30 | 24 | [M-COO]- | PC36:4 (16:0d9-20:4) |
| PC40:4(20:0_20:4) | 882.6 | 303.2 | N | 30 | 24 | [M-COO]- | PC36:4 (16:0d9-20:4) |
| PC40:5(18:0_22:5) | 880.6 | 329.2 | N | 30 | 24 | [M-COO]- | PC36:4 (16:0d9-20:4) |
| PC40:5(18:1_22:4) | 880.6 | 331.3 | N | 30 | 24 | [M-COO]- | PC36:4 (16:0d9-20:4) |
| PC40:5(20:0_20:5) | 880.6 | 301.2 | N | 30 | 24 | [M-COO]- | PC36:4 (16:0d9-20:4) |
| PC40:6(18:0_22:6) | 878.6 | 327.2 | N | 30 | 24 | [M-COO]- | PC36:4 (16:0d9-20:4) |
| PC40:6(18:1_22:5) | 878.6 | 329.2 | N | 30 | 24 | [M-COO]- | PC36:4 (16:0d9-20:4) |
| PC40:6(18:2_22:4) | 878.6 | 331.3 | N | 30 | 24 | [M-COO]- | PC36:4 (16:0d9-20:4) |
| PC40:7(18:1_22:6) | 876.6 | 327.2 | N | 30 | 24 | [M-COO]- | PC36:4 (16:0d9-20:4) |
| PC40:7(18:2_22:5) | 876.6 | 329.2 | N | 30 | 24 | [M-COO]- | PC36:4 (16:0d9-20:4) |
| PC40:7(20:3_20:4) | 876.6 | 305.2 | N | 30 | 24 | [M-COO]- | PC36:4 (16:0d9-20:4) |
| PC40:8(18:2_22:6) | 874.5 | 327.2 | N | 30 | 24 | [M-COO]- | PC36:4 (16:0d9-20:4) |
| PC42:10(20:4_22:6) | 898.6 | 327.2 | N | 30 | 24 | [M-COO]- | PC36:4 (16:0d9-20:4) |
| PC42:6(20:0_22:6) | 906.6 | 327.2 | N | 30 | 24 | [M-COO]- | PC36:4 (16:0d9-20:4) |
| PC(P30:0) (P16:0_14:0) | 734.5 | 227.2 | N | 30 | 24 | [M-COO]- | PC(P32:0) (P16:0_16:0d9) |
| PC(P32:0) (P16:0_16:0) | 762.6 | 255.2 | N | 30 | 24 | [M-COO]- | PC(P32:0) (P16:0_16:0d9) |
| PC(P32:0) (P16:0_16:1) | 760.6 | 253.2 | N | 30 | 24 | [M-COO]- | PC(P32:0) (P16:0_16:0d9) |
| PC(P34:0) (P16:0_18:0) | 790.6 | 283.3 | N | 30 | 24 | [M-COO]- | PC(P32:0) (P16:0_16:0d9) |
| PC(P34:0) (P18:0_16:0) | 790.6 | 255.2 | N | 30 | 24 | [M-COO]- | PC(P32:0) (P16:0_16:0d9) |
| PC(P34:1) (P16:0_18:1) | 788.6 | 281.3 | N | 30 | 24 | [M-COO]- | PC(P32:0) (P16:0_16:0d9) |
| PC(P34:1) (P18:0_16:1) | 788.6 | 253.2 | N | 30 | 24 | [M-COO]- | PC(P32:0) (P16:0_16:0d9) |
| PC(P34:1) (P18:1_16:0) | 788.6 | 255.2 | N | 30 | 24 | [M-COO]- | PC(P32:0) (P16:0_16:0d9) |
| PC(P34:2) (P16:0_18:2) | 786.6 | 279.2 | N | 30 | 24 | [M-COO]- | PC(P32:0) (P16:0_16:0d9) |
| PC(P34:3) (P16:0_18:3) | 784.6 | 277.2 | N | 30 | 24 | [M-COO]- | PC(P32:0) (P16:0_16:0d9) |
| PC(P36:1) (P18:0_18:1) | 816.6 | 281.3 | N | 30 | 24 | [M-COO]- | PC(P32:0) (P16:0_16:0d9) |
| PC(P36:1) (P18:1_18:0) | 816.6 | 283.3 | N | 30 | 24 | [M-COO]- | PC(P32:0) (P16:0_16:0d9) |
| PC(P36:2) (P18:0_18:2) | 814.6 | 279.2 | N | 30 | 24 | [M-COO]- | PC(P32:0) (P16:0_16:0d9) |
| PC(P36:2) (P18:1_18:1) | 814.6 | 281.3 | N | 30 | 24 | [M-COO]- | PC(P32:0) (P16:0_16:0d9) |
| PC(P36:3) (P16:0_20:3) | 812.6 | 305.2 | N | 30 | 24 | [M-COO]- | PC(P32:0) (P16:0_16:0d9) |
| PC(P36:3) (P18:0_18:3) | 812.6 | 277.2 | N | 30 | 24 | [M-COO]- | PC(P32:0) (P16:0_16:0d9) |
| PC(P36:4) (P16:0_20:4) | 810.6 | 303.2 | N | 30 | 24 | [M-COO]- | PC(P32:0) (P16:0_16:0d9) |
| PC(P36:5) (P16:0_20:5) | 808.6 | 301.2 | N | 30 | 24 | [M-COO]- | PC(P32:0) (P16:0_16:0d9) |
| PC(P38:0) (P20:0_18:0) | 846.7 | 283.3 | N | 30 | 24 | [M-COO]- | PC(P32:0) (P16:0_16:0d9) |
| PC(P38:2) (P20:0_18:2) | 842.6 | 279.2 | N | 30 | 24 | [M-COO]- | PC(P32:0) (P16:0_16:0d9) |
| PC(P38:3) (P18:0_20:3) | 840.6 | 305.2 | N | 30 | 24 | [M-COO]- | PC(P32:0) (P16:0_16:0d9) |
| PC(P38:4) (P16:0_22:4) | 838.6 | 331.2 | N | 30 | 24 | [M-COO]- | PC(P32:0) (P16:0_16:0d9) |
| PC(P38:4) (P18:0_20:4) | 838.6 | 303.2 | N | 30 | 24 | [M-COO]- | PC(P32:0) (P16:0_16:0d9) |
| PC(P38:5) (P18:0_20:5) | 836.6 | 301.2 | N | 30 | 24 | [M-COO]- | PC(P32:0) (P16:0_16:0d9) |
| PC(P38:6) (P16:0_22:6) | 834.6 | 327.2 | N | 30 | 24 | [M-COO]- | PC(P32:0) (P16:0_16:0d9) |
| PC(P40:4) (P18:0_22:4) | 866.6 | 331.2 | N | 30 | 24 | [M-COO]- | PC(P32:0) (P16:0_16:0d9) |
| PC(P40:4) (P20:0_20:4) | 866.6 | 303.2 | N | 30 | 24 | [M-COO]- | PC(P32:0) (P16:0_16:0d9) |
| PC(P40:5) (P20:0_20:5) | 864.6 | 301.2 | N | 30 | 24 | [M-COO]- | PC(P32:0) (P16:0_16:0d9) |
| PC(P40:6) (P18:0_22:6) | 862.6 | 327.2 | N | 30 | 24 | [M-COO]- | PC(P32:0) (P16:0_16:0d9) |
| PE(P34:1) P-16:0_18:1 | 700.5 | 281.2 | N | 30 | 24 | [M-H]- | PE(P32:0) (P16:0_16:0d9) |
| PE(P34:2) P-16:0_18:2 | 698.5 | 279.2 | N | 30 | 24 | [M-H]- | PE(P32:0) (P16:0_16:0d9) |
| PE(P36:1) P-18:0_18:1 | 728.5 | 281.2 | N | 30 | 24 | [M-H]- | PE(P32:0) (P16:0_16:0d9) |
| PE(P36:2) P-18:0_18:2 | 726.5 | 279.2 | N | 30 | 24 | [M-H]- | PE(P32:0) (P16:0_16:0d9) |
| PE(P36:2) P-18:1_18:1 | 726.5 | 281.2 | N | 30 | 24 | [M-H]- | PE(P32:0) (P16:0_16:0d9) |
| PE(P36:3) P-18:1_18:2 | 724.5 | 279.2 | N | 30 | 24 | [M-H]- | PE(P32:0) (P16:0_16:0d9) |
| PE(P36:4) P-16:0_20:4 | 722.5 | 303.2 | N | 30 | 24 | [M-H]- | PE(P32:0) (P16:0_16:0d9) |
| PE(P36:5) P16:0_20:5 | 720.5 | 301.2 | N | 30 | 24 | [M-H]- | PE(P32:0) (P16:0_16:0d9) |
| PE(P38:2) P-20:0_18:2 | 754.6 | 279.2 | N | 30 | 24 | [M-H]- | PE(P32:0) (P16:0_16:0d9) |
| PE(P38:3) P-18:0_20:3 | 752.5 | 305.2 | N | 30 | 24 | [M-H]- | PE(P32:0) (P16:0_16:0d9) |
| PE(P38:4) P-16:0_22:4 | 750.5 | 331.2 | N | 30 | 24 | [M-H]- | PE(P32:0) (P16:0_16:0d9) |
| PE(P38:4) P-18:0_20:4 | 750.5 | 303.2 | N | 30 | 24 | [M-H]- | PE(P32:0) (P16:0_16:0d9) |
| PE(P38:5) P-16:0_22:5 | 748.5 | 329.2 | N | 30 | 24 | [M-H]- | PE(P32:0) (P16:0_16:0d9) |
| PE(P38:5) P-18:0_20:5 | 748.5 | 301.2 | N | 30 | 24 | [M-H]- | PE(P32:0) (P16:0_16:0d9) |
| PE(P38:5) P-18:1_20:4 | 748.5 | 303.2 | N | 30 | 24 | [M-H]- | PE(P32:0) (P16:0_16:0d9) |
| PE(P38:6) P-16:0_22:6 | 746.7 | 327.2 | N | 30 | 24 | [M-H]- | PE(P32:0) (P16:0_16:0d9) |
| PE(P40:4) P-18:0_22:4 | 778.6 | 331.3 | N | 30 | 24 | [M-H]- | PE(P32:0) (P16:0_16:0d9) |
| PE(P40:4) P-20:0_20:4 | 778.6 | 303.2 | N | 30 | 24 | [M-H]- | PE(P32:0) (P16:0_16:0d9) |
| PE(P40:4) P-20:1_20:3 | 778.6 | 305.2 | N | 30 | 24 | [M-H]- | PE(P32:0) (P16:0_16:0d9) |
| PE(P40:5) P-18:0_22:5 | 776.5 | 329.2 | N | 30 | 24 | [M-H]- | PE(P32:0) (P16:0_16:0d9) |
| PE(P40:5) P-20:0_20:5 | 776.5 | 301.2 | N | 30 | 24 | [M-H]- | PE(P32:0) (P16:0_16:0d9) |
| PE(P40:5) P-20:1_20:4 | 776.5 | 303.2 | N | 30 | 24 | [M-H]- | PE(P32:0) (P16:0_16:0d9) |
| PE(P40:6) P-18:0_22:6 | 774.7 | 327.2 | N | 30 | 24 | [M-H]- | PE(P32:0) (P16:0_16:0d9) |
| PE(P40:7) P-18:1_22:6 | 772.7 | 327.2 | N | 30 | 24 | [M-H]- | PE(P32:0) (P16:0_16:0d9) |
| PE30:1(16:0_14:0) | 662.5 | 255.2 | N | 30 | 24 | [M-H]- | PE32:0(16:0_16:0d9) |
| PE32:0(16:0_16:0) | 690.5 | 255.2 | N | 30 | 24 | [M-H]- | PE32:0(16:0_16:0d9) |
| PE32:1(14:0_18:1) | 688.5 | 281.2 | N | 30 | 24 | [M-H]- | PE32:0(16:0_16:0d9) |
| PE32:1(16:0_16:1) | 688.5 | 253.2 | N | 30 | 24 | [M-H]- | PE32:0(16:0_16:0d9) |
| PE32:1(18:0_14:1) | 688.5 | 225.2 | N | 30 | 24 | [M-H]- | PE32:0(16:0_16:0d9) |
| PE32:2(14:0_18:2) | 686.5 | 279.2 | N | 30 | 24 | [M-H]- | PE32:0(16:0_16:0d9) |
| PE33:0(18:0_15:0) | 704.5 | 241.2 | N | 30 | 24 | [M-H]- | PE32:0(16:0_16:0d9) |
| PE34:0(18:0_16:0) | 718.5 | 283.3 | N | 30 | 24 | [M-H]- | PE32:0(16:0_16:0d9) |
| PE34:1(16:0_18:1) | 716.5 | 281.2 | N | 30 | 24 | [M-H]- | PE32:0(16:0_16:0d9) |
| PE34:1(18:0_16:1) | 716.5 | 283.3 | N | 30 | 24 | [M-H]- | PE32:0(16:0_16:0d9) |
| PE34:2(16:0_18:2) | 714.5 | 279.2 | N | 30 | 24 | [M-H]- | PE32:0(16:0_16:0d9) |
| PE34:2(18:1_16:1) | 714.5 | 281.2 | N | 30 | 24 | [M-H]- | PE32:0(16:0_16:0d9) |
| PE34:3(14:0_20:3) | 712.5 | 305.2 | N | 30 | 24 | [M-H]- | PE32:0(16:0_16:0d9) |
| PE34:3(16:0_18:3) | 712.5 | 277.2 | N | 30 | 24 | [M-H]- | PE32:0(16:0_16:0d9) |
| PE34:3(18:2_16:1) | 712.5 | 279.2 | N | 30 | 24 | [M-H]- | PE32:0(16:0_16:0d9) |
| PE34:4(14:0_20:4) | 710.5 | 303.2 | N | 30 | 24 | [M-H]- | PE32:0(16:0_16:0d9) |
| PE34:5(14:0_20:5) | 708.5 | 301.2 | N | 30 | 24 | [M-H]- | PE32:0(16:0_16:0d9) |
| PE35:0(18:0_17:0) | 732.6 | 269.2 | N | 30 | 24 | [M-H]- | PE32:0(16:0_16:0d9) |
| PE36:0(18:0_18:0) | 746.6 | 283.3 | N | 30 | 24 | [M-H]- | PE32:0(16:0_16:0d9) |
| PE36:1(16:0_20:1) | 744.6 | 309.3 | N | 30 | 24 | [M-H]- | PE32:0(16:0_16:0d9) |
| PE36:1(18:0_18:1) | 744.6 | 281.2 | N | 30 | 24 | [M-H]- | PE32:0(16:0_16:0d9) |
| PE36:2(16:0_20:2) | 742.5 | 307.3 | N | 30 | 24 | [M-H]- | PE32:0(16:0_16:0d9) |
| PE36:2(18:0_18:2) | 742.5 | 279.2 | N | 30 | 24 | [M-H]- | PE32:0(16:0_16:0d9) |
| PE36:2(18:1_18:1) | 742.5 | 281.2 | N | 30 | 24 | [M-H]- | PE32:0(16:0_16:0d9) |
| PE36:3(16:0_20:3) | 740.5 | 305.2 | N | 30 | 24 | [M-H]- | PE32:0(16:0_16:0d9) |
| PE36:3(18:0_18:3) | 740.5 | 277.2 | N | 30 | 24 | [M-H]- | PE32:0(16:0_16:0d9) |
| PE36:3(18:1_18:2) | 740.5 | 279.2 | N | 30 | 24 | [M-H]- | PE32:0(16:0_16:0d9) |
| PE36:4(16:0_20:4) | 738.5 | 303.2 | N | 30 | 24 | [M-H]- | PE32:0(16:0_16:0d9) |
| PE36:4(18:1_18:3) | 738.5 | 277.2 | N | 30 | 24 | [M-H]- | PE32:0(16:0_16:0d9) |
| PE36:4(18:2_18:2) | 738.5 | 279.2 | N | 30 | 24 | [M-H]- | PE32:0(16:0_16:0d9) |
| PE36:5(16:0_20:5) | 736.5 | 301.2 | N | 30 | 24 | [M-H]- | PE32:0(16:0_16:0d9) |
| PE36:6(14:0_22:6) | 734.5 | 327.2 | N | 30 | 24 | [M-H]- | PE32:0(16:0_16:0d9) |
| PE37:1(15:0_22:1) | 758.6 | 241.2 | N | 30 | 24 | [M-H]- | PE32:0(16:0_16:0d9) |
| PE37:2(17:0_20:2) | 756.6 | 269.2 | N | 30 | 24 | [M-H]- | PE32:0(16:0_16:0d9) |
| PE37:4(17:0_20:4) | 752.5 | 269.2 | N | 30 | 24 | [M-H]- | PE32:0(16:0_16:0d9) |
| PE38:2(18:0_20:2) | 770.6 | 307.3 | N | 30 | 24 | [M-H]- | PE32:0(16:0_16:0d9) |
| PE38:2(18:1_20:1) | 770.6 | 309.3 | N | 30 | 24 | [M-H]- | PE32:0(16:0_16:0d9) |
| PE38:3(18:0_20:3) | 768.6 | 305.2 | N | 30 | 24 | [M-H]- | PE32:0(16:0_16:0d9) |
| PE38:3(18:1_20:2) | 768.6 | 307.3 | N | 30 | 24 | [M-H]- | PE32:0(16:0_16:0d9) |
| PE38:3(18:2_20:1) | 768.6 | 309.3 | N | 30 | 24 | [M-H]- | PE32:0(16:0_16:0d9) |
| PE38:4(16:0_22:4) | 766.5 | 331.3 | N | 30 | 24 | [M-H]- | PE32:0(16:0_16:0d9) |
| PE38:4(18:0_20:4) | 766.5 | 303.2 | N | 30 | 24 | [M-H]- | PE32:0(16:0_16:0d9) |
| PE38:4(18:1_20:3) | 766.5 | 305.2 | N | 30 | 24 | [M-H]- | PE32:0(16:0_16:0d9) |
| PE38:5(16:0_22:5) | 764.5 | 329.2 | N | 30 | 24 | [M-H]- | PE32:0(16:0_16:0d9) |
| PE38:5(18:0_20:5) | 764.5 | 301.2 | N | 30 | 24 | [M-H]- | PE32:0(16:0_16:0d9) |
| PE38:5(18:1_20:4) | 764.5 | 303.2 | N | 30 | 24 | [M-H]- | PE32:0(16:0_16:0d9) |
| PE38:5(18:2_20:3) | 764.5 | 305.2 | N | 30 | 24 | [M-H]- | PE32:0(16:0_16:0d9) |
| PE38:6(16:0_22:6) | 762.5 | 327.2 | N | 30 | 24 | [M-H]- | PE32:0(16:0_16:0d9) |
| PE38:6(18:1_20:5) | 762.5 | 301.2 | N | 30 | 24 | [M-H]- | PE32:0(16:0_16:0d9) |
| PE38:6(18:2_20:4) | 762.5 | 303.2 | N | 30 | 24 | [M-H]- | PE32:0(16:0_16:0d9) |
| PE38:7(18:2_20:5) | 760.5 | 301.2 | N | 30 | 24 | [M-H]- | PE32:0(16:0_16:0d9) |
| PE39:5(17:0_22:5) | 778.5 | 269.2 | N | 30 | 24 | [M-H]- | PE32:0(16:0_16:0d9) |
| PE40:1(22:0_18:1) | 800.6 | 281.2 | N | 30 | 24 | [M-H]- | PE32:0(16:0_16:0d9) |
| PE40:4(18:0_22:4) | 794.6 | 331.3 | N | 30 | 24 | [M-H]- | PE32:0(16:0_16:0d9) |
| PE40:5(18:0_22:5) | 792.6 | 329.2 | N | 30 | 24 | [M-H]- | PE32:0(16:0_16:0d9) |
| PE40:5(18:1_22:4) | 792.6 | 331.3 | N | 30 | 24 | [M-H]- | PE32:0(16:0_16:0d9) |
| PE40:6(18:0_22:6) | 790.5 | 327.2 | N | 30 | 24 | [M-H]- | PE32:0(16:0_16:0d9) |
| PE40:6(18:1_22:5) | 790.5 | 329.2 | N | 30 | 24 | [M-H]- | PE32:0(16:0_16:0d9) |
| PE40:6(18:2_22:4) | 790.5 | 331.3 | N | 30 | 24 | [M-H]- | PE32:0(16:0_16:0d9) |
| PE40:6(18:2_22:6) | 786.5 | 327.2 | N | 30 | 24 | [M-H]- | PE32:0(16:0_16:0d9) |
| PE40:7(18:1_22:6) | 788.5 | 327.2 | N | 30 | 24 | [M-H]- | PE32:0(16:0_16:0d9) |
| PE40:7(18:2_22:5) | 788.5 | 329.2 | N | 30 | 24 | [M-H]- | PE32:0(16:0_16:0d9) |
| PI32:0(16:0_16:1) | 809.5 | 255.2 | N | 30 | 24 | [M-H]- | PE32:0(16:0_16:0d9) |
| PI34:1(16:0_18:1) | 833.5 | 281.3 | N | 30 | 24 | [M-H]- | PE32:0(16:0_16:0d9) |
| PI34:2(16:0_18:2) | 831.4 | 255.2 | N | 30 | 24 | [M-H]- | PE32:0(16:0_16:0d9) |
| PI36:1(18:0_18:1) | 863.5 | 281.2 | N | 30 | 24 | [M-H]- | PE32:0(16:0_16:0d9) |
| PI36:3(16:0_20:3) | 859.6 | 305.2 | N | 30 | 24 | [M-H]- | PE32:0(16:0_16:0d9) |
| PI36:3(18:1_18:2) | 859.6 | 281.2 | N | 30 | 24 | [M-H]- | PE32:0(16:0_16:0d9) |
| PI36:4(16:0_20:4) | 857.6 | 255.2 | N | 30 | 24 | [M-H]- | PE32:0(16:0_16:0d9) |
| PI38:4(18:0_20:4) | 879.5 | 283.3 | N | 30 | 24 | [M-H]- | PE32:0(16:0_16:0d9) |
| PI38:5(18:1_20:4) | 877.6 | 281.3 | N | 30 | 24 | [M-H]- | PE32:0(16:0_16:0d9) |
| PI40:5(18:0_22:5) | 889.6 | 329.2 | N | 30 | 24 | [M-H]- | PE32:0(16:0_16:0d9) |
| PI40:6(16:0_22:6) | 887.5 | 327.2 | N | 30 | 24 | [M-H]- | PE32:0(16:0_16:0d9) |
| PS34:2 (16:1_18:1) | 758.5 | 253.2 | N | 30 | 24 | [M-H]- | PE32:0(16:0_16:0d9) |
| PS34:3 (16:1_18:2) | 756.5 | 279.3 | N | 30 | 24 | [M-H]- | PE32:0(16:0_16:0d9) |
| PS36:0 (18:0_18:0) | 790.6 | 283.3 | N | 30 | 24 | [M-H]- | PE32:0(16:0_16:0d9) |
| PS36:1 (18:0_18:1) | 788.5 | 283.3 | N | 30 | 24 | [M-H]- | PE32:0(16:0_16:0d9) |
| PS36:1 (20:1_16:0) | 788.5 | 309.3 | N | 30 | 24 | [M-H]- | PE32:0(16:0_16:0d9) |
| PS36:2 (18:0_18:2) | 786.5 | 279.2 | N | 30 | 24 | [M-H]- | PE32:0(16:0_16:0d9) |
| PS36:2 (18:1_18:1) | 786.5 | 281.3 | N | 30 | 24 | [M-H]- | PE32:0(16:0_16:0d9) |
| PS36:2 (20:1_16:1) | 786.5 | 309.3 | N | 30 | 24 | [M-H]- | PE32:0(16:0_16:0d9) |
| PS36:2 (20:2_16:0) | 786.5 | 307.3 | N | 30 | 24 | [M-H]- | PE32:0(16:0_16:0d9) |
| PS36:3 (18:0_18:3) | 784.5 | 283.2 | N | 30 | 24 | [M-H]- | PE32:0(16:0_16:0d9) |
| PS36:3 (18:1_18:2) | 784.5 | 279.2 | N | 30 | 24 | [M-H]- | PE32:0(16:0_16:0d9) |
| PS38:2 (18:0_20:2) | 814.6 | 307.3 | N | 30 | 24 | [M-H]- | PE32:0(16:0_16:0d9) |
| PS38:4 (18:0_20:4) | 810.5 | 283.3 | N | 30 | 24 | [M-H]- | PE32:0(16:0_16:0d9) |
| PS38:5 (18:0_20:5) | 808.5 | 301.2 | N | 30 | 24 | [M-H]- | PE32:0(16:0_16:0d9) |
| PS40:3 (20:0_20:3) | 840.6 | 311.3 | N | 30 | 24 | [M-H]- | PE32:0(16:0_16:0d9) |
| PS40:4 (20:0_20:4) | 838.6 | 311.3 | N | 30 | 24 | [M-H]- | PE32:0(16:0_16:0d9) |
| PS40:5 (20:0_20:5) | 836.5 | 311.3 | N | 30 | 24 | [M-H]- | PE32:0(16:0_16:0d9) |
| PS40:5 (20:1_20:4) | 836.5 | 309.3 | N | 30 | 24 | [M-H]- | PE32:0(16:0_16:0d9) |
| PS40:6 (18:0_22:6) | 834.5 | 327.2 | N | 30 | 24 | [M-H]- | PE32:0(16:0_16:0d9) |
| PS40:6 (20:1_20:5) | 834.5 | 309.3 | N | 30 | 24 | [M-H]- | PE32:0(16:0_16:0d9) |
| PS40:6 (20:2_20:4) | 834.5 | 307.3 | N | 30 | 24 | [M-H]- | PE32:0(16:0_16:0d9) |
| PS42:5 (20:1_22:4) | 864.6 | 309.3 | N | 30 | 24 | [M-H]- | PE32:0(16:0_16:0d9) |
| PS42:6 (20:0_22:6) | 862.6 | 311.3 | N | 30 | 24 | [M-H]- | PE32:0(16:0_16:0d9) |
| PS42:7 (20:1_22:6) | 860.5 | 309.3 | N | 30 | 24 | [M-H]- | PE32:0(16:0_16:0d9) |
| PS38:3 (18:0_20:3) | 812.5 | 305.3 | N | 30 | 24 | [M-H]- | PE32:0(16:0_16:0d9) |
| PS38:4 (16:0_22:4) | 810.5 | 331.2 | N | 30 | 24 | [M-H]- | PE32:0(16:0_16:0d9) |
| PS38:4 (18:1_20:3) | 810.5 | 305.2 | N | 30 | 24 | [M-H]- | PE32:0(16:0_16:0d9) |
| PS38:5 (18:1_20:4) | 808.5 | 281.2 | N | 30 | 24 | [M-H]- | PE32:0(16:0_16:0d9) |
| PS38:5 (18:2_20:3) | 808.5 | 305.2 | N | 30 | 24 | [M-H]- | PE32:0(16:0_16:0d9) |
| Cer34:0(d18:0_c16:0) | 538.5 | 280.3 | N | 30 | 24 | [M-H]- | PE32:0(16:0_16:0d9) |
| Cer34:1(d16:1_c18:0) | 536.5 | 308.3 | N | 30 | 24 | [M-H]- | PE32:0(16:0_16:0d9) |
| Cer34:1(d18:1_c16:0) | 536.5 | 280.3 | N | 30 | 24 | [M-H]- | PE32:0(16:0_16:0d9) |
| Cer34:2(d18:2_c16:0) | 534.5 | 280.3 | N | 30 | 24 | [M-H]- | PE32:0(16:0_16:0d9) |
| Cer36:1(d16:1_c20:0) | 564.5 | 336.3 | N | 30 | 24 | [M-H]- | PE32:0(16:0_16:0d9) |
| Cer36:1(d18:1_c18:0) | 564.5 | 308.3 | N | 30 | 24 | [M-H]- | PE32:0(16:0_16:0d9) |
| Cer38:1(d16:1_c22:0) | 592.6 | 364.4 | N | 30 | 24 | [M-H]- | PE32:0(16:0_16:0d9) |
| Cer38:1(d18:1_c20:0) | 592.6 | 336.4 | N | 30 | 24 | [M-H]- | PE32:0(16:0_16:0d9) |
| Cer40:0(d18:0_c22:0) | 622.6 | 364.4 | N | 30 | 24 | [M-H]- | PE32:0(16:0_16:0d9) |
| Cer40:1(d16:1_c24_0) | 620.6 | 392.4 | N | 30 | 24 | [M-H]- | PE32:0(16:0_16:0d9) |
| Cer40:1(d18:1_c22:0) | 620.6 | 364.4 | N | 30 | 24 | [M-H]- | PE32:0(16:0_16:0d9) |
| Cer40:2(d16:1_c24:1) | 618.6 | 390.4 | N | 30 | 24 | [M-H]- | PE32:0(16:0_16:0d9) |
| Cer40:2(d18:2_c22:0) | 618.6 | 364.4 | N | 30 | 24 | [M-H]- | PE32:0(16:0_16:0d9) |
| Cer41:0(d18:0_c23:0) | 636.3 | 378.4 | N | 30 | 24 | [M-H]- | PE32:0(16:0_16:0d9) |
| Cer41:1(d17:1_c24:0) | 634.6 | 392.4 | N | 30 | 24 | [M-H]- | PE32:0(16:0_16:0d9) |
| Cer41:1(d18:1_c23:0) | 634.6 | 378.4 | N | 30 | 24 | [M-H]- | PE32:0(16:0_16:0d9) |
| Cer41:2(d17:1_c24:1) | 632.6 | 390.4 | N | 30 | 24 | [M-H]- | PE32:0(16:0_16:0d9) |
| Cer41:2(d18:2_c23:0) | 632.6 | 378.4 | N | 30 | 24 | [M-H]- | PE32:0(16:0_16:0d9) |
| Cer42:0(d18:0_c24:0) | 650.6 | 392.4 | N | 30 | 24 | [M-H]- | PE32:0(16:0_16:0d9) |
| Cer42:1(d18:0_c24:1) | 648.6 | 390.4 | N | 30 | 24 | [M-H]- | PE32:0(16:0_16:0d9) |
| Cer42:1(d18:1_c24:0) | 648.6 | 392.5 | N | 30 | 24 | [M-H]- | PE32:0(16:0_16:0d9) |
| Cer42:2(d18:1_c24:1) | 646.6 | 390.5 | N | 30 | 24 | [M-H]- | PE32:0(16:0_16:0d9) |
| Cer42:3(d18:2_c24:1) | 644.6 | 390.4 | N | 30 | 24 | [M-H]- | PE32:0(16:0_16:0d9) |
| SM(14:0) | 675.5 | 184.1 | P | 30 | 40 | [M+NH4]+ | TAG(52:4) 16:0_20:4_16:0d9 |
| SM(16:0) | 703.6 | 184.1 | P | 30 | 40 | [M+NH4]+ | TAG(52:4) 16:0_20:4_16:0d9 |
| SM(18:0) | 731.6 | 184.1 | P | 30 | 40 | [M+NH4]+ | TAG(52:4) 16:0_20:4_16:0d9 |
| SM(18:1) | 729.6 | 184.1 | P | 30 | 40 | [M+NH4]+ | TAG(52:4) 16:0_20:4_16:0d9 |
| SM(20:0) | 759.6 | 184.1 | P | 30 | 40 | [M+NH4]+ | TAG(52:4) 16:0_20:4_16:0d9 |
| SM(20:1) | 757.6 | 184.1 | P | 30 | 40 | [M+NH4]+ | TAG(52:4) 16:0_20:4_16:0d9 |
| SM(22:0) | 787.7 | 184.1 | P | 30 | 40 | [M+NH4]+ | TAG(52:4) 16:0_20:4_16:0d9 |
| SM(22:1) | 785.7 | 184.1 | P | 30 | 40 | [M+NH4]+ | TAG(52:4) 16:0_20:4_16:0d9 |
| SM(24:0) | 815.7 | 184.1 | P | 30 | 40 | [M+NH4]+ | TAG(52:4) 16:0_20:4_16:0d9 |
| SM(24:1) | 813.7 | 184.1 | P | 30 | 40 | [M+NH4]+ | TAG(52:4) 16:0_20:4_16:0d9 |
| SM(26:0) | 843.7 | 184.1 | P | 30 | 40 | [M+NH4]+ | TAG(52:4) 16:0_20:4_16:0d9 |
| SM(26:1) | 841.7 | 184.1 | P | 30 | 40 | [M+NH4]+ | TAG(52:4) 16:0_20:4_16:0d9 |
